# Probing the stereoselectivity of OleD-catalyzed glycosylation of cardiotonic steroids†

**DOI:** 10.1039/c7ra11979h

**Published:** 2018-01-30

**Authors:** Xue-Lin Zhu, Chao Wen, Qing-Mei Ye, Wei Xu, Deng-Lang Zou, Guang-Ping Liang, Fan Zhang, Wan-Na Chen, Ren-Wang Jiang

**Affiliations:** Guangdong Province Key Laboratory of Pharmacodynamic Constituents of TCM and New Drugs Research, College of Pharmacy, Jinan University Guangzhou 510632 P. R. China trwjiang@jnu.edu.cn; Department of Pharmacy, Hainan General Hospital Haikou 570311 P. R. China

## Abstract

The glycosyltransferase OleD variant as a catalyst for the glycosylation of four pairs of epimers of cardiotonic steroids (CTS) are assessed. The results of this study demonstrated that the OleD-catalyze glycosylation of CTS is significantly influenced by the configuration at C-3 and the A/B fusion mode. 3β-OH and A/B ring *cis* fusion are favoured by OleD (ASP). An epoxide ring at C-14 and C-15 further increases the bioconversion rate; while an acetyl group at C-16 and lactone ring type at C-17 did not influence the biotransformation. A high conversion rate corresponded to a low *K*_m_ value. A molecular docking simulation showed that filling of hydrophobic pocket II and interaction with residue Tyr115 may play an important role in the glycosylation reactions catalyzed by OleD glycosyltransferases. Furthermore, the glycosylation products showed a stronger inhibitory activity for Na^+^, K^+^-ATPase than the corresponding aglycones. This study provides the first stereoselective properties for OleD (ASP) catalyzed glycosylation.

## Abstract 2

The glycosyltransferase OleD variant as a catalyst for the glycosylation of four pairs of epimers of cardiotonic steroids (CTS) was assessed.

## Graphical abstract

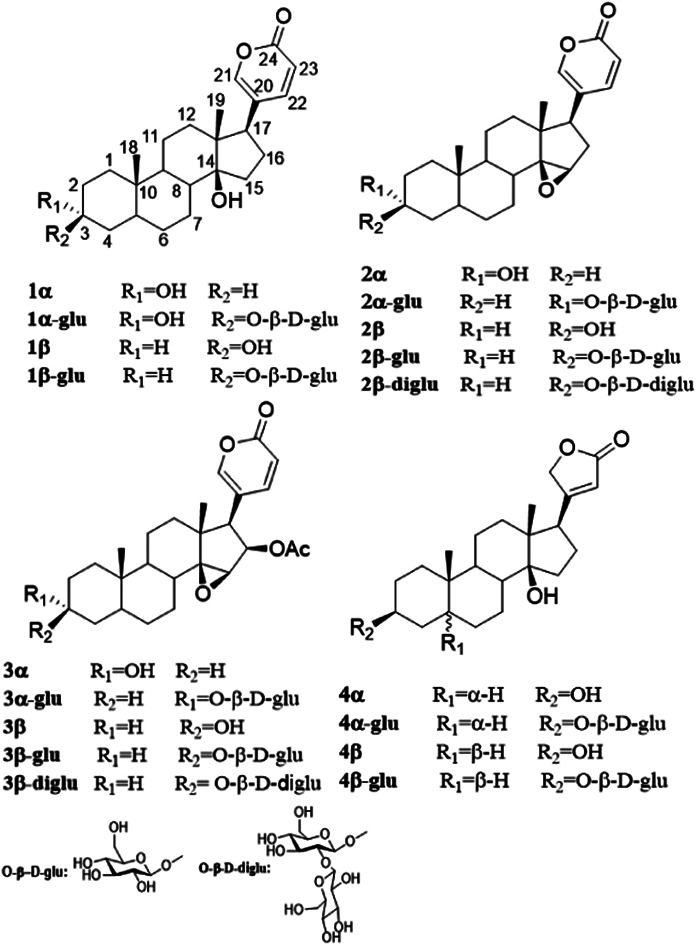

## Introduction

The glycosyltransferase OleD from *Streptomyces antibioticus* catalyzes the glycosylation of oleandomycin using UDP-D-glucose (UDPG) as the glycosyl donor.^1–4^ Recent studies revealed an enhanced triple mutant (A242V/S132F/P67T, OleD (ASP)) that displayed marked improvement in proficiency and substrate promiscuity. The OleD (ASP) was found to show highly permissive properties and is capable of glycosylation on 100 diverse acceptors, *e.g.* erythromycin,^5^ flavones,^6^ indole alkaloids^7^ and steroids.^8^ Nowadays, 56% of the drugs in clinics are chiral compounds; however 88% of them are marketed as racemates consisting of an equimolar mixture of two enantiomers.^8^ Although OleD (ASP) was found to catalyze the glycosylation of cardenolide and bufadienolide aglycone with a bias toward the desired C-3 regiospecificity,^7^ the stereospecificity of this enzyme remains unreported.

Cardiotonic steroids (CTS)^9,10^ such as digitoxin, digoxin and proscillaridin are clinically important drugs for treatment of congestive heart failure for more than two centuries due to their potent inhibition of Na^+^, K^+^-ATPase (NKA), which is an integral membrane protein maintaining ionic gradients in all superior eukaryotic cells.^11–15^ Natural CTS includes cardenolides and bufadienolides. Cardenolides, such as ouabain and digoxin, possess a five-membered lactone ring at position C-17β of the steroidal skeleton. The natural cardenolide can be aglycone or glycosides with one or more sugar groups at C-3.^16,17^ Bufadienolides, isolated from many plants and animals such as *Bufo bufo gargarizans*, bears a six-membered lactone ring at C-17β. The bufadienolides can also be aglycone or glycosides in plants but only aglycone in animals.^18–21^ For both bufadienolides and cardenolides, there is a hydroxy group at C-3, either β- or α-configuration. Normally the CTS with a 3β-OH shows more remarkable activities than the 3α-diastereomer.^22^ Especially, the sugar groups of C-3 of CTS play a key role for the inhibitory activity of NKA.^23^

In this paper, we use OleD (ASP) to catalyze the glycosylation of four pairs of epimers of cardiotonic steroids. *K*_m_ values of two pairs of epimers representing the bufadienolides and cardenolides were determined. Molecular docking was used to simulate the interactions between the substrate and enzyme. Finally, the inhibitory activities against NKA of CTS aglycone and the corresponding glycosides were compared.

## Experimental section

### General experimental procedures

All chemicals and reagents were purchased from Sigma unless otherwise stated. The NMR spectra were recorded on a Bruker AV-400 spectrophotometer (Bruker, Germany) with TMS as internal standard. Chemical shifts (*δ*) were expressed in ppm with reference to the solvent signals. HR-ESI-MS were determined on a Micromass Q-TOF mass spectrometer (Waters, USA). Analytical HPLC is run on Agilent 1200 system (Agilent, USA) with a Phenomenex Luna C-18 column (250 mm × 4.6 mm, 5 μm, USA). Preparative HPLC was performed on a Wu Feng HPLC system (Shanghai, China) equipped with a preparative reverse phase column (20 × 250 mm, 5 μm).

### Cloning and expression of variant OleD (ASP) glycosyltransferase

The OleD (ASP) glycosyltransferase gene was generated by Genscript Biotechnology (Nanjing, China) and was cloned into DH5α (Takara, Japan) and pET28a (Novagen, USA) expression vector. Single colony of *Escherichia coli* BL21 (DE3) pLysS (Tiangen, China) transformed with pET28a/OleD vector was inoculated in Luria Bertani (LB) medium (3 mL) supplemented with 50 μg mL^−1^ kanamycin. The medium was cultured overnight at 37 °C with shaking (200 rpm). The entire starter culture was then transferred to 1 L LB medium supplemented with 50 μg mL^−1^ kanamycin and grown at 37 °C with shaking (200 rpm) until the OD_600_ reached 0.6. Isopropyl β-d-thiogalactoside (IPTG) was subsequently added (final concentration to 0.4 mM) and the culture was incubated at 18 °C for 18 h. Then the cell pellets were collected by centrifugation at 10 000*g* at 4 °C for 20 min and the supernatant was discarded.^24^ Pellets were resuspended in 10 mL PBS buffer (20 mM phosphate buffer, 0.2 M NaCl, 2 mM KCl, pH 7.4) and then lysed by sonication. Cell debris was removed by centrifugation at 10 000*g* at 4 °C for 20 min and the clear supernatant was immediately submitted to His60 Ni super flow resin (Clontech, USA). The resin was balanced by the equilibration buffer (50 mM sodium phosphate buffer, 0.3 M NaCl, 20 mM imidazole, pH 7.4). The enzyme was allowed to bind for 1 h at 4 °C with gentle agitation, and the resin was washed with 10 mL mild buffer (20 mM phosphate buffer, 0.5 M NaCl, 50 mM imidazole, pH 7.4). Finally, the protein was eluted by with 20 mL strong buffer (50 mM sodium phosphate buffer, 0.3 M NaCl, 300 mM imidazole, pH 7.4). The enzyme aliquots were immediately frozen in liquid nitrogen and stored at −80 °C. Protein purity was confirmed by SDS-PAGE to be >95% and protein concentration for all studies was determined using the Bradford Protein Assay Kit from Bio-Rad (TransGen Biotech, China).

### Preparation of epimers of bufadienolides

CTS (1β, 2β and 3β, 10.7 mM) was dissolved in CH_2_Cl_2_ (35 mL) in a round bottom flask. Pyridinium chloride hydrochloride (PCC) (21.4 mM) was added and stirred constantly to dissolve. The solution was stirred for 2 h at room temperature. Then the solvent was removed under reduced pressure and the residue was dissolved in anhydrous tetrahydrofuran (5 mL) in a round bottom flask. Sodium borohydride (NaBH_4_) was added and stirred constantly to dissolve. The solution was stirred for 1 h at room temperature. Then 5 mL of water was added slowly to the reaction solution. Ethyl acetate (10 mL) was used to extract the mixture and the solvent was removed under reduced pressure. The final residue was purified by preparative HPLC eluting with acetonitrile to yield C-3α CTS (1α, 2α, 3α).

1α: ^1^H NMR (CD_3_OD, 400 MHz) *δ*: 8.01 (1H, dd, *J* = 9.7, 2.5 Hz, H-22), 7.45 (1H, d, *J* = 2.4 Hz, H-21), 6.30 (1H, d, *J* = 9.8 Hz, H-23), 3.58 (1H, m, H-3), 2.58 (1H, m, H-17), 2.26–2.11 (2H, m), 1.92–1.62 (11H, m), 1.55–1.36 (9H, m), 1.29–1.19 (1H, m), 1.09–1.01 (2H, m), 0.95 (3H, s, H-19), 0.73 (3H, s, H-18); ^13^C NMR (CD_3_OD, 100 MHz) *δ*: 164.8 (C-24), 150.5 (C-21), 149.37 (C-22), 125.0 (C-20), 115.4 (C-23), 86.1 (C-14), 72.3 (C-3), 52.3 (C-17), 49.8 (C-13), 43.2 (C-9), 43.1 (C-5), 41.9 (C-12), 37.7 (C-8), 37.0 (C-4), 36.3 (C-1), 35.9 (C-10), 33.2 (C-15), 31.3 (C-2), 29.9 (C-16), 28.4 (C-6), 23.8 (C-19), 22.8 (C-7), 22.5 (C-11), 17.3 (C-18); HR-ESI-MS: *m*/*z* 387.2576 [M + H]^+^ (calcd for C_24_H_34_O_4_, 387.2534).

2α: ^1^H NMR (CD_3_OD, 400 MHz) *δ*: 7.91 (1H, dd, *J* = 9.8, 2.4 Hz, H-22), 7.47 (1H, d, *J* = 2.4 Hz, H-21), 6.28 (1H d, *J* = 9.8 Hz, H-23), 3.62 (1H, s, H-15), 3.57 (1H, m, H-3), 2.61 (1H, d, *J* = 10.0 Hz, H-10), 2.44 (1H, d, *J* = 10.4 Hz, H-16), 2.04–1.63 (9H, m), 1.63–1.28 (9H, m), 0.99 (3H, s), 0.79 (3H, s); ^13^C NMR (CD_3_OD, 100 MHz) *δ*: 164.5 (C-24), 151.8 (C-21), 149.6 (C-22), 124.5 (C-20), 115.3 (C-23), 75.8 (C-14), 72.2 (C-3), 61.1 (C-15), 48.6 (C-17), 46.3 (C-13), 43.1 (C-5), 41.3 (C-9), 40.1 (C-12), 36.9 (C-4), 36.1 (C-1), 36.0 (C-10), 35.2 (C-8), 33.2 (C-16), 31.3 (C-2), 27.5 (C-6), 23.8 (C-19), 22.1 (C-11), 22.0 (C-7), 17.1 (C-18); HR-ESI-MS: *m*/*z* 385.2363 [M + H]^+^ (calcd for C_24_H_32_O_4_, 385.2407).

3α: ^1^H NMR (CD_3_OD, 400 MHz) *δ*: 8.04 (1H, d, *J* = 9.6 Hz, H-22), 7.39 (1H, s, H-21), 6.26 (1H, d, *J* = 9.6 Hz, H-23), 5.51 (1H, d, *J* = 10.3 Hz, H-16), 3.76 (1H, s, H-15), 3.58 (1H, m, H-3), 2.95 (1H, d, *J* = 9.3 Hz, H-17), 2.20–2.01 (1H, td, H-8), 1.81 (3H, s, COCH_3_), 1.79–1.04 (15H, m), 0.99 (3H, s, H-19), 0.83 (3H, s, H-18); ^13^C NMR (CD_3_OD, 100 MHz) *δ*: 171.6 (C̲OCH_3_), 164.0 (C-24), 153.5 (C-22), 150.9 (C-21), 118.4 (C-20), 114.1 (C-23), 76.6 (C-16), 73.4 (C-14), 72.2 (C-3), 60.8 (C-15), 51.4 (C-17), 46.3 (C-13), 43.0 (C-5), 41.1 (C-9), 40.7 (C-12), 36.9 (C-4), 36.1 (C-1), 36.0 (C-10), 34.7 (C-8), 31.2 (C-2), 27.4 (C-6), 23.7 (C-19), 21.9 (C-7), 21.9 (C-11), 20.4 (COC̲H_3_), 17.5 (C-18); HR-ESI-MS: *m*/*z* 443.2445 [M + H]^+^ (calcd for C_26_H_34_O_6_, 443.2314).

### General pilot-scale reaction

*In vitro* glycosylation reactions were carried out in 500 μL reaction buffer (50 mM Tris–HCl, pH 8.0) containing 250 μg of enzyme, 2.5 mM of UDPG, 1 mM aglycon and 5 mM MgCl_2_. The mixture was incubated at 37 °C for 16 h. The reaction mixture was subsequently frozen and lyophilized, and the residue was resuspended in 500 μL ice cold methanol and filtered. One portion of each clarified reaction mixture was analyzed by analytical reverse-phase HPLC equipped with a Phenomenex Luna-C18 column (250 mm × 4.6 mm, 5 μm). The flow rate was 1 mL min^−1^. The gradient was consisted of solvent A (0.1% trifluoroacetic acid/H_2_O) and B (100% acetonitrile): (a) 0–20 min, 10–75% B; (b) 20–21 min, 75–100% B; (c) 21–26 min, 100% B; (d) 26–29 min, 100–10% B; and (e) 29–35 min, 10% B. Detections were set at 220 and 296 nm. Conversion rate was calculated by the corresponding HPLC peak area percentage using the Agilent Chromatography Workstation Software. The LC-ESI-MS analysis was accomplished using standard C-18 reversed-phase chromatography with diode array detection wherein 5% of the flow was diverted to time-of-flight (TOF) mass spectrometer.

### Preparative scale glycosylation reaction

Aglycons (10 mg) were dissolved in 5% of DMSO and transferred to pH 8 buffer solution (50 mM Tris–HCl, 5 mM MgCl_2_). UDPG was added followed by OleD (ASP) catalyst. After 16 h incubation at 37 °C, the reaction was stopped with equal volume of ice cold methanol. Then the reaction mixture was centrifuged at 10 000*g* for 30 min and the supernatant was concentrated under reduced pressure, and the debris was resuspended in 5 mL of ice-cold methanol and filtered with 0.22 μM membrane. The filtrate was subjected to preparative HPLC (20 × 250 mm, 5 μm; flow rate: 5 mL min^−1^; *A*_296_ and *A*_220_ detection) using water/acetonitrile as the eluent to afford the corresponding of glucosides. The compound was then characterized using high resolution MS, ^1^D and ^2^D NMR, including ^1^H, ^13^C and HSQC.

#### 1β-glu

^1^H NMR (CD_3_OD, 400 MHz) *δ*: 8.03 (1H, dd, *J* = 9.7, 2.3 Hz, H-22), 7.45 (1H, d, *J* = 1.8 Hz, H-21), 6.30 (1H, d, *J* = 9.6 Hz, H-23), 4.37 (1H, d, *J* = 7.8 Hz, sugar H-1), 4.12 (1H, m, H-3), 3.88 (1H, d, *J* = 11.9, 1.8 Hz), 3.71 (1H, dd, *J* = 5.4 Hz), 3.40–3.20 (4H, m), 2.56–2.52 (1H, m, H-17), 2.26–2.11 (2H, m), 1.92–1.22 (23H, m), 1.09–1.01 (2H, m), 0.95 (3H, s, H-19), 0.73 (3H, s, H-18); ^13^C NMR (CD_3_OD, 100 MHz) *δ*: 164.8 (C-24), 150.5 (C-21), 149.37 (C-22), 125.0 (C-20), 115.4 (C-23), 102.7 (sugar C-1), 86.1 (C-14), 78.2 (sugar C-5), 77.84 (sugar C-2), 75.6 (sugar C-4), 75.2 (sugar C-3), 71.8 (C-3), 61.7 (sugar C-6), 52.3 (C-17), 49.8 (C-13), 43.2 (C-9), 43.1 (C-5), 41.9 (C-12), 37.7 (C-8), 37.0 (C-4), 36.3 (C-1), 35.9 (C-10), 33.2 (C-15), 31.3 (C-2), 29.9 (C-16), 28.4 (C-6), 23.8 (C-19), 22.8 (C-7), 22.5 (C-11), 17.3 (C-18); HR-ESI-MS: *m*/*z* 549.3035 [M + H]^+^ (calcd for C_30_H_44_O_9_, 549.3021).

#### 2β-glu

^1^H NMR (CD_3_OD, 400 MHz) *δ*: 7.91 (1H, dd, *J* = 9.8, 2.4 Hz, H-22), 7.46 (1H, d, *J* = 2.4 Hz, H-21), 6.27 (1H d, *J* = 9.8 Hz, H-23), 4.32 (1H, d, *J* = 7.8 Hz, sugar H-1), 4.08 (1H, m, H-3), 3.86 (1H, d, *J* = 11.9 Hz), 3.67 (1H, dd, *J* = 11.9, 5.4 Hz), 3.62 (1H, s, H-15), 3.39–3.17 (7H, m), 2.61 (1H, d, *J* = 10.0 Hz, H-10), 2.44 (1H, d, *J* = 10.4 Hz, H-16), 2.04–1.43 (15H, m), 1.40–1.05 (4H, m), 0.99 (3H, s), 0.79 (3H, s); ^13^C NMR (CD_3_OD, 100 MHz) *δ*: 164.5 (C-24), 151.8 (C-21), 149.6 (C-22), 124.5 (C-20), 115.3 (C-23), 102.7 (sugar C-1), 78.2 (sugar C-5), 77.84 (sugar C-2), 75.8 (C-14), 75.4 (sugar C-4), 75.2 (sugar C-3), 71.7 (C-3), 62.8 (sugar C-6), 61.2 (C-15), 48.6 (C-17), 46.3 (C-13), 40.7 (C-12), 40.1 (C-9), 37.4 (C-10), 36.3 (C-5), 35.0 (C-4), 33.2 (C-8), 31.1 (C-1), 30.8 (C-16), 27.5 (C-2), 27.0 (C-6), 24.1 (C-19), 22.2 (C-11), 21.7 (C-7), 17.2 (C-18); HR-ESI-MS: *m*/*z* 547.2902 [M + H]^+^ (calcd for C_30_H_42_O_9_, 547.2938).

#### 3β-glu

^1^H NMR (CD_3_OD, 400 MHz) *δ*: 7.91 (1H, dd, *J* = 9.8, 2.4 Hz, H-22), 7.47 (1H, d, *J* = 2.4 Hz, H-21), 6.28 (1H d, *J* = 9.8 Hz, H-23), 4.34 (1H, d, *J* = 7.8 Hz, sugar H-1), 4.10 (1H, m, H-3), 3.87 (1H, dd, *J* = 11.8, 1.8 Hz), 3.76 (1H, s, H-15), 3.69 (1H, dd, *J* = 11.8, 5.4 Hz), 3.58 (1H, m, H-3), 3.45–3.17 (5H, m), 2.95 (1H, d, *J* = 9.3 Hz, H-17), 2.15–2.01 (1H, td, H-8), 1.85 (3H, s, COCH_3_), 1.81–1.04 (19H, m), 0.99 (3H, s, H-19), 0.83 (3H, s, H-18); ^13^C NMR (CD_3_OD, 100 MHz) *δ*: 171.6 (C̲OCH_3_), 164.0 (C-24), 153.5 (C-22), 150.9 (C-21), 118.4 (C-20), 114.1 (C-23), 102.7 (sugar C-1), 78.2 (sugar C-5), 77.84 (sugar C-2), 76.6 (C-16), 75.4 (sugar C-4), 75.2 (sugar C-3), 73.4 (C-14), 71.7 (C-3), 62.8 (sugar C-6), 60.8 (C-15), 51.4 (C-17), 46.3 (C-13), 40.5 (C-5), 40.7 (C-9), 37.3 (C-12), 36.3 (C-4), 36.1 (C-1), 36.0 (C-10), 31.7 (C-8), 31.2 (C-2), 27.4 (C-6), 23.7 (C-19), 21.9 (C-7), 21.9 (C-11), 20.4 (COC̲H_3_), 17.5 (C-18). HR-ESI-MS: *m*/*z* 605.2996 [M + H]^+^ (calcd for C_32_H_44_O_11_, 605.2938).

#### 4β-glu

^1^H NMR (CD_3_OD, 400 MHz) *δ*: 5.93 (1H, s), 5.07 (1H, d, *J* = 20, 18.4 Hz), 4.95 (1H, d, *J* = 19.8 Hz), 4.35 (1H, d, *J* = 7.8 Hz, sugar H-1), 4.11 (1H, s, H-3), 3.89 (1H, d, *J* = 2.1 Hz), 3.69 (1H, dd, *J* = 11.8, 5.4 Hz), 3.40–3.21 (5H, m), 2.95–2.79 (1H, m), 2.35–2.12 (2H, m), 2.00–1.43 (19H, m), 1.40–1.22 (4H, m), 1.00 (3H, s), 0.92 (3H, s); ^13^C NMR (CD_3_OD, 100 MHz) *δ*: 178.5 (C-23), 177.3 (C-20), 117.8 (C-22), 102.7 (sugar C-1), 86.5 (C-14), 78.2 (sugar C-5), 77.8 (sugar C-2), 75.4 (sugar C-4), 75.3 (sugar C-3), 75.2 (C-31), 71.7 (C-3), 62.8 (sugar C-6), 52.2 (C-13), 51.1 (C-17), 42.7 (C-9), 41.0 (C-8), 37.5 (C-5), 36.9 (C-12), 36.3 (C-10), 33.4 (C-4), 31.2 (C-15), 30.9 (C-1), 28.1 (C-2), 27.8 (C-6), 27.5 (C-16), 24.1 (C-7), 22.6 (C-7), 22.4 (C-18), 16.4 (C-19); HR-ESI-MS: *m*/*z* 537.8049 [M + H]^+^ (calcd for C_29_H_44_O_9_, 537.8021).

### Determination of kinetic parameters

Assays were performed in a final volume of 200 μL 50 mM Tris–HCl (pH 8.0), and contained constant concentrations of OleD (ASP) (40 μg) and UDPG (2.5 mM) while varying the concentration (0.01–1.2 mM) of 2β, 2α, 4β and 4α. Aliquots (100 μL) were removed every 15 min, mixed with an equal volume of ice cold methanol, and centrifuged at 10 000*g* for 10 min. Supernatants were analyzed by analytical reverse-phase HPLC. Conversion rate is calculated by the corresponding HPLC peak area percentage using the Agilent Chromatography Workstation Software.^25^ All experiments were performed in triplicate. Initial velocities were fitted to the Michaelis–Menten equation using Origin Pro 7.0 software.

### Molecular docking

The program Autodock Vina was used for docking simulations. For docking purpose, the crystal structure of OleD (PDBID 4M60) was retrieved from Protein Data Bank. To create receptor and ligand structures for docking, the following procedure was conducted. Firstly, the 3D structures of the ligands were prepared using the Gaussian 09 program at the B3LYP/6-31G(d) level. Harmonic vibration frequencies were calculated to confirm the stability of these conformers. Then the receptor and optimized structure of the ligands were converted to required pdbqt format using Autodock Tools 1.5.4. The Autodock Vina parameters were set as follow, box size: 15 × 15 × 15 Å, the center of box: *x* = 38.96, *y* = 61.05, *z* = 13.83, the exhaustiveness: 100, and number of output conformations was set to 20. The calculated geometries were ranked in terms of free energy of binding and the best poses were selected for further analysis. All molecular visualizations were carried out in PyMOL software.

### Assessment inhibition NKA activity of CTS

The inhibitory effects of CTS on NKA were determined essentially as previously reported method.^11,26,27^

## Results and discussion

### Preparation of epimers of bufadienolides

Three epimers, *i.e.* α-bufalin (1α), α-resibufogenin (2α) and α-cinobufagin (3α) were synthesized from β-bufalin (1β), β-resibufogenin (3β) and β-cinobufagin (3β), respectively, by an oxidation with pyridinium chloride hydrochloride followed by a reduction with sodium borohydride. Uzarigenin (4α) and digitoxigenin (4β), two natural cardenolides with a *trans* and *cis* A/B fusion mode (4α, 4βFig. 1), respectively, were identified from the whole herb of *Asclepias curassavica*^28^ and the roots of *Streptocaulon juventas*,^29^ respectively. These probes are suitable for us to investigate whether the configurations at C-3 and the fusion mode of A/B ring influence the OleD-catalyze glycosylation.

**Fig. 1:**
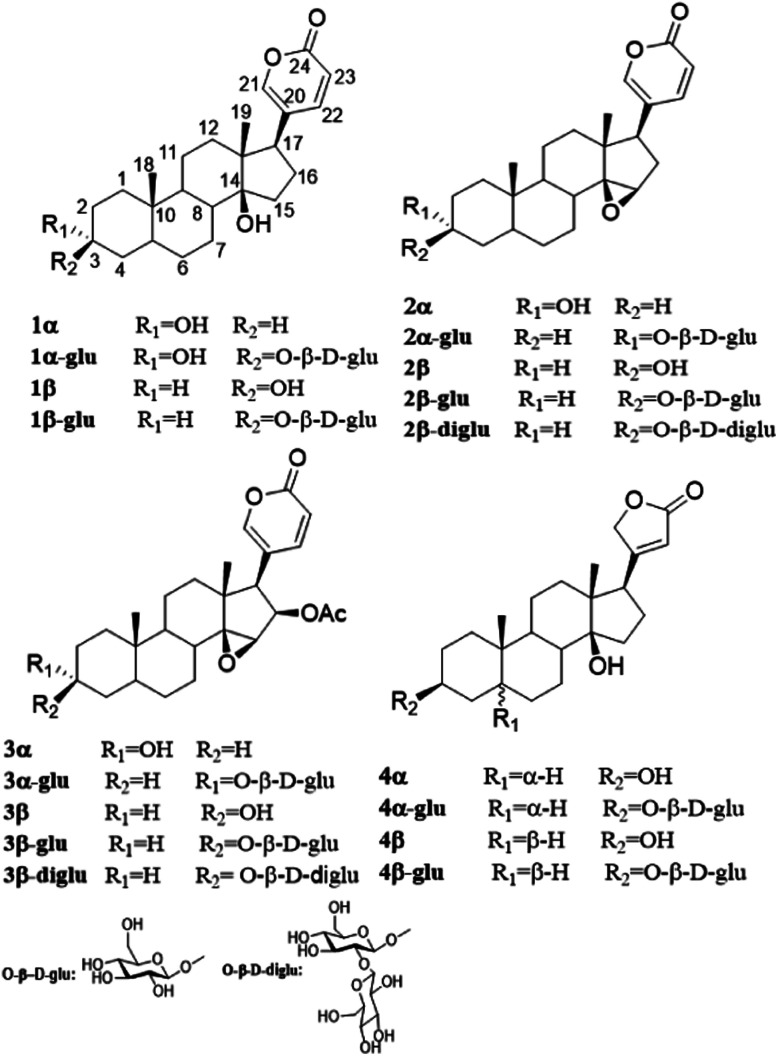
Structures of cardiotonic steroid substrates and the corresponding glycosylation products.

### Glycosylation of 1β and 1α

Both 3α- and 3β-hydroxylated bufalin are endogenous in *Bufo bufo gargarizans*. Though only 3β-hydroxylated bufalin is present as a toxic chemical defence in toad venom, both epimers were found to occur at a 2 : 3 ratio in the heart and a 1 : 2 ratio in the blood.^22^ 3β-Hydroxylated bufalin is the major active component of the toad venom and exhibits potent cardiotonic activity.^30,31^ Its structural core includes a *cis*–*trans*–*cis* fused steroid core with two hydroxyl groups at C-3 and C-14. However, it has serious toxicity because there is no distinct selectivity for α1 and α2 subunits of NKA. Fortunately, a lot of bufalin analogues including glycosides have been prepared by chemical and biological transformation. Recently, our group reported synthesis of 3β-*N*-methoxy-*N*-β-d-glucoside of bufalin which could enhance its inhibition on NKA.^22^ The latest crystal structure demonstrated that the level of glycosylation affect the depth of CTS binding and that the steroid core substituents fine tune the configuration of transmembrane helices αM1-2.^27^ Furthermore, the sugar unit could enhance the selectivity on the alpha2 isoform of NKA; however, chemical synthesis of bufalin glycoside was laborious and consumed a lot of toxic reagents. It is necessary to develop a green method for glycosylation of bufadienolides.

**Fig. 2:**
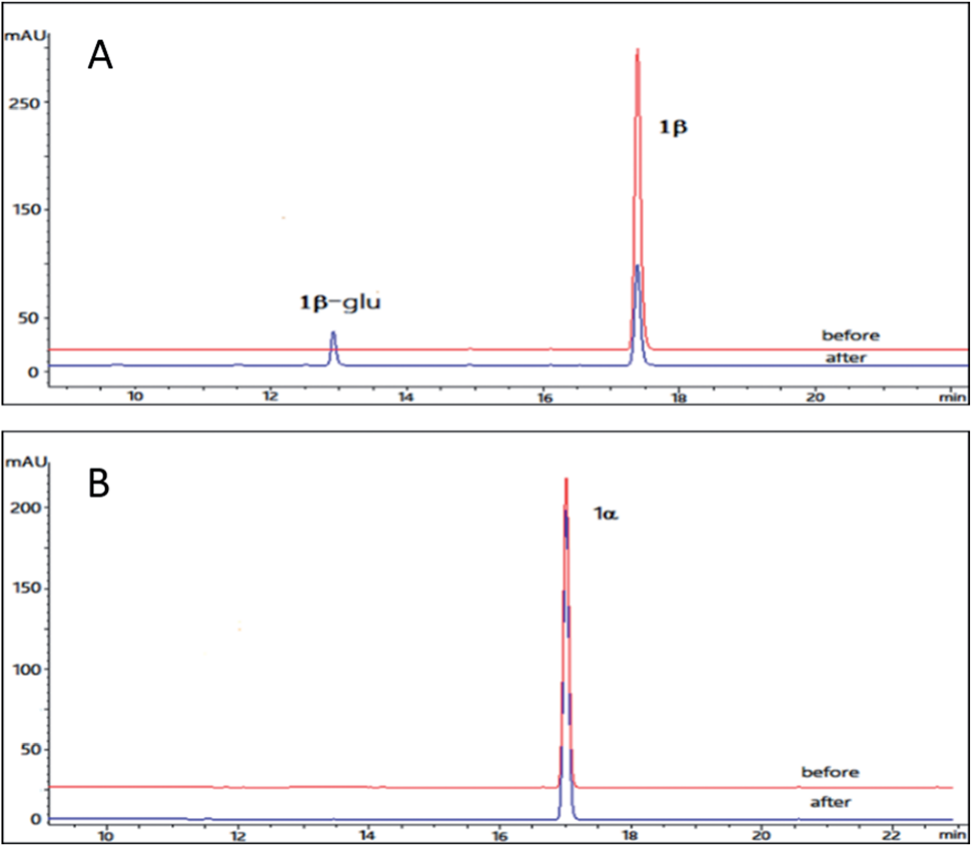
HPLC chromatograms of the enzymatic reactions. (A) 1β without UDPG and OleD (ASP) (before); 1β with UDPG and OleD (ASP) (after); (B) 1α without UDPG and OleD (ASP) (before) (1); 1α with UDPG and OleD (ASP) (after).

The pilot biotransformation of 1β and 1α were carried out using UDPG as the sugar donor and OleD (ASP) as the catalyst under the standard conditions (0.5 mM UDPG, 0.1 mM aglycon, 16 h).^5^ The result of LC-MS showed that OleD (ASP) could catalyze the generation of monoglycoside of 1β with a conversion rate 30%; while glycosylation product of 1α was not detected (Fig. 2). Thus the bioconversion of 1β and 1α catalyzed by OleD (ASP) was influenced by the C-3 configuration. The conversion rates of different isomers were compared in Table 1.

**Table 1:** Conversion rates of individual compounds

Compounds	Products	Conversion rate (%)
1α	1α-glu	N. T^a^
1β	1β-glu	30
2α	2α-glu	1
2β	2β-diglu	1
	2β-glu	79
3α	3α-glu	2
3β	3β-diglu	5
	3β-glu	70
4α	4α-glu	2
4β	4β-glu	25

a N. T: not detected.

To maximize the production of bufalin-3-*O*-β-d-glucoside (1β-glu), a 20 h reaction was carried out at 37 °C. Compound 1β (10 mg, 40 mM) was dissolved in DMSO (0.625 mL) and diluted with buffer solution (50 mM Tris–HCl, 5 mM MgCl_2_, pH 8.0, 25 mL total volume). UDPG (38 mg, 50 mM) was added along with OleD (ASP) (13 mg). The reaction was stopped with 25 mL of ice cold methanol. Then the reaction mixture was centrifuged at 10 000*g* for 30 min and supernatant was concentrated under reduced pressure. The residue was dissolved in 3 mL methanol, and filtered with 0.22 μM membrane. The filtrate was subjected to preparative HPLC using water/acetonitrile as the eluent. Finally, 3.1 mg of 1β-glu was obtained with a conversion rate of 21%. The structure of 1β-glu was determined by ^1^D NMR and HR-ESI-MS analysis. The pseudo molecular ion *m*/*z* 549.3035 [M + H]^+^ in the HR-ESI-MS was corresponding to a formula C_30_H_44_O_9_ (calcd for 549.3021), which was 162 unit larger than the parent compound bufalin, suggesting the formation of monoglucoside. Compared with 1β, the chemical shift *δ*_C-3_ of 1β-glu was shifted to downfield by 3.9 ppm and the *δ*_H-3_ was shifted to downfield by 0.02 ppm; while C-14 was unchanged, confirming the glycosylation site was at 3-OH. The β-configuration was determined by the large coupling constant of the anomeric proton (*δ*_H_ 4.37, d, *J* = 7.8 Hz). Based on the above analysis, it was concluded that OleD (ASP) could catalyze the transformation of 1β to its monoglucoside 1β-glu at 3-OH.

### Glycosylation of 2β and 2α

Resibufogenin (2β) is another type of bufadienolides from the venom of *Bufo bufo gargarizans* with an 14,15-epoxide ring in contrast to the 14-OH in bufalin. Compound 2β has been reported to exhibit a wide range of activities such as cardiotonic, renal sodium excretion, blood pressure stimulating, antitumor activity and immunoregulatory activity.^32^ However, the application of resibufogenin is restricted because of its strong toxicity.^33^ In the past several years, several structure modifications on 2β was performed which generate more than 30 derivatives.^23^ In order to obtain the glycosylation derivative of 2β and compare the NKA inhibitory activities of these derivatives with different configurations, OleD (ASP) catalysed biotransformation on 2β and 2α were carried out.

**Fig. 3:**
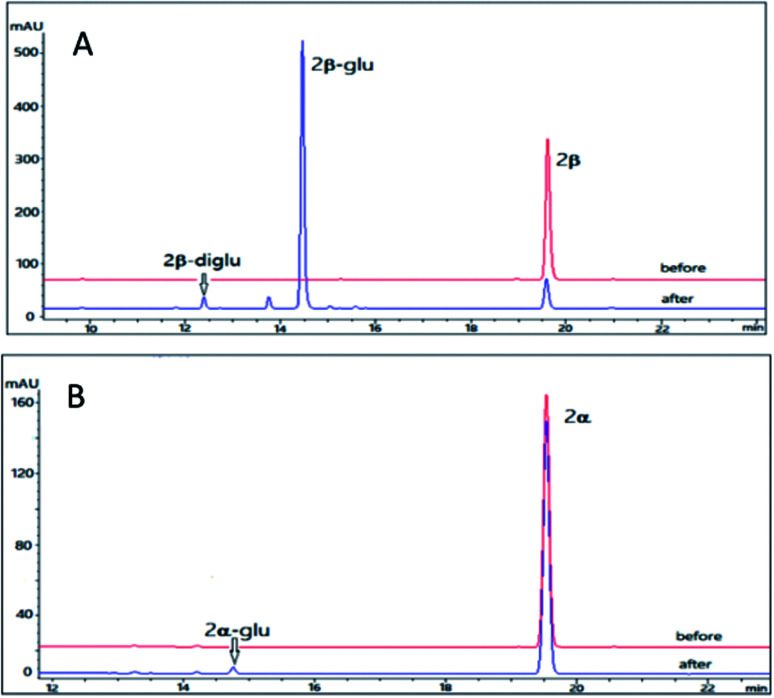
HPLC chromatograms of the enzymatic reactions. (A) 2β without UDPG and OleD (ASP) (before); 2β with UDPG and OleD (ASP) (after); (B) 2α without UDPG and OleD (ASP) (before); 2α with UDPG and OleD (ASP) (after).

The pilot reaction conditions are the same as those described for 1β and 1α. According to the result of LC-MS analysis, OleD (ASP) catalyzed the formation of monoglucoside (major) and diglucoside (minor) of 2β with a total conversion rate 80% which was much higher than that of 1β; while the conversion rate of monoglucoside 2α-glu is about 1% in contrast to the absence of 1α glucoside (Fig. 3). Thus similar to 1β and 1α, the configuration at C-3 was also important for conversion rate of 2β and 2α. The much high conversion rate of 2β and 2α might be due to the 14,15-epoxide moiety as compared to the 14-OH for 1β and 1α.

To maximize the production of resibufogenin-3-*O*-β-d-glucoside (2β-glu), a preparative scale experiment was undertaken. Using the same condition as 1β, while 2β (10 mg, 40 mM) was served as substrate, we obtain 8.4 mg 2β-glu with a conversion rate 65%. The identification of compounds 2β-glu was determined by ^1^D NMR and HR-ESI-MS analysis. The pseudo-molecular ion *m*/*z* 547.2902 [M + H]^+^ in the HR-ESI-MS corresponded to a formula C_30_H_42_O_9_ (calcd for 547.2938) which was 162 unit larger than the parent compound 2β. Compared with 2β, the chemical shift C-3 of 2β-glu was shifted to downfield by 3.9 ppm and the chemical shift H-3 was shifted to downfield by 0.02 ppm, confirming the glycosylation site was also at 3-OH. The β-configuration was determined by the large coupling constant of the anomeric proton (*δ*_H_ 4.32, d, *J* = 7.8 Hz). Accordingly, it was concluded that OleD (ASP) could catalyze the transformation of 2β to its glucoside 2β-glu with a much higher rate than 1β. It is noteworthy that bioconversion of 2β also lead to the generation of a diglucoside (2β-diglu) which was confirm by the HR-MS *m*/*z* 709.3421 [M + H]^+^. Due to the small amount, it was not determined by NMR.

### Glycosylation of 3β and 3α

Cinobufagin is also a major bufadienolides (4–6% dry weight) from the venom of *Bufo bufo gargarizans* with an 14,15-epoxide ring and an acetyl group at C-16.^34^ Cinobufagin was found to show potent cardiotonic, blood pressure-stimulating, antiviral, local anesthetic and antineoplastic activities.^34,35^ However, the poor water solubility restricted its clinical use. Though a series of analogues of cinobufagin has been generated by chemical synthesis and cell suspension cultures,^33,36^ the glycosylation method is rarely reported. Furthermore, difference between the glycosidation profile on the 3β and 3α isomers of cinobufagin is unclear.

**Fig. 4:**
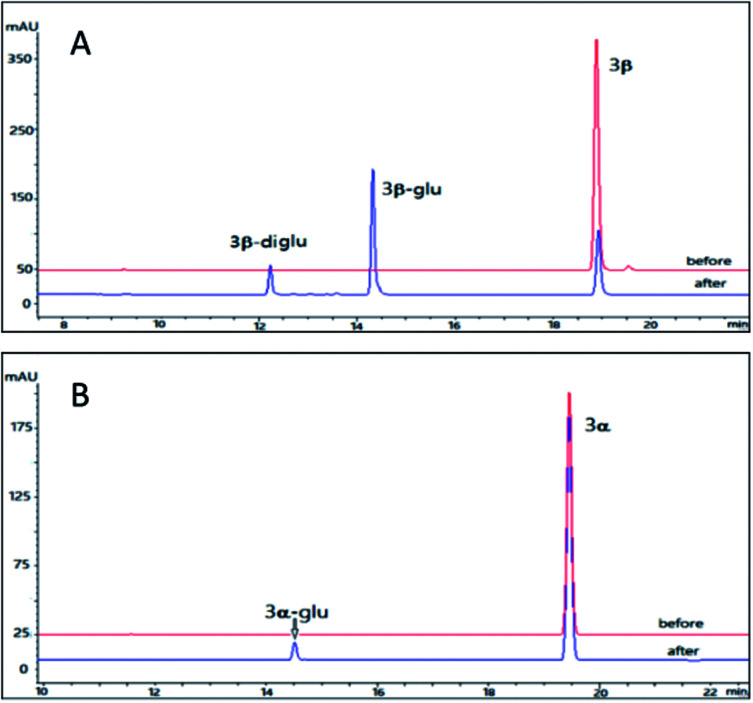
HPLC chromatograms of the enzymatic reactions. (A) 3β without UDPG and OleD (ASP) (before); 3β with UDPG and OleD (ASP) (after); (B) 3α without UDPG and OleD (ASP) (before); 3α with UDPG and OleD (ASP) (after).

Using the same reaction conditions as described for 2β and 2α, OleD (ASP) was found to catalyze the glycosylation of 3β leading to both monoglucoside (3β-glu) and diglucoside (3β-diglu) of 3β with a total conversion rate of 75%; while at the same condition as 3β, only the monoglucoside of (3α-glu) was detected with a low conversion rate 2% (Fig. 4). Similarly, this phenomenon further confirmed the importance of configuration at C-3 for the conversion rate. It is noteworthy that with an additional acetyl group at C-16 compound 3β showed similar conversion rate as 2β. Similarly, 3β also generate a diglucoside (3β-diglu) which was confirm by the HR-ESI-MS *m*/*z* 767.3479 [M + H]^+^. Due to the small amount, it was not determined by NMR.

Using the same conditions as 2β, we prepared 6.9 mg 3β-glu with a conversion rate 50% (10 mg 3β was served as substrate). The identification of the product 3β-glu was also confirmed by ^1^D and ^2^D NMR and HR-ESI-MS analysis. HR-ESI-MS showed a pseudo molecular ion *m*/*z* 605.2996 [M + H]^+^ corresponding to a formula C_32_H_44_O_11_ (calcd for 605.2938) which was 162 unit larger than the parent compound 3β. Similar to 2β-glu, the chemical shifts of C-3 and H-3 of 3β-glu were shifted to downfield by 3.9 ppm and 0.02 ppm, respectively, confirming the glycosylation site at 3-OH. The β-configuration was also confirmed by the large coupling constant of the anomeric proton (*δ*_H_ 4.32, d, *J* = 7.8 Hz). Accordingly, it was concluded that OleD (ASP) could catalyze the transformation of 3β into 3β-glu.

### Glycosylation of 4β and 4α

As compared to the three pairs of bufadienolides 1β and 1α, 2β and 2α, 3β and 3α, digitoxigenin (4β) and uzarigenin (4α) were two cardenolides with the same substitution pattern. The only difference between 4β and 4α is the A/B ring fusion modes, which are *cis* and *trans*, respectively. Compound 4β was the aglycone of digoxin, a commonly used cardiotonic drug.^37^ Recently, it is reported that introduction of a sugar unit at C-3 of cardenolides could improves NKA isoform selectivity (α2/α3 over α1).^38,39^ In order to compare the glycosylation profiles of 4β and 4α with different A/B ring fusion mode, the OleD (ASP) catalyzed bioconversion was carried out.

**Fig. 5:**
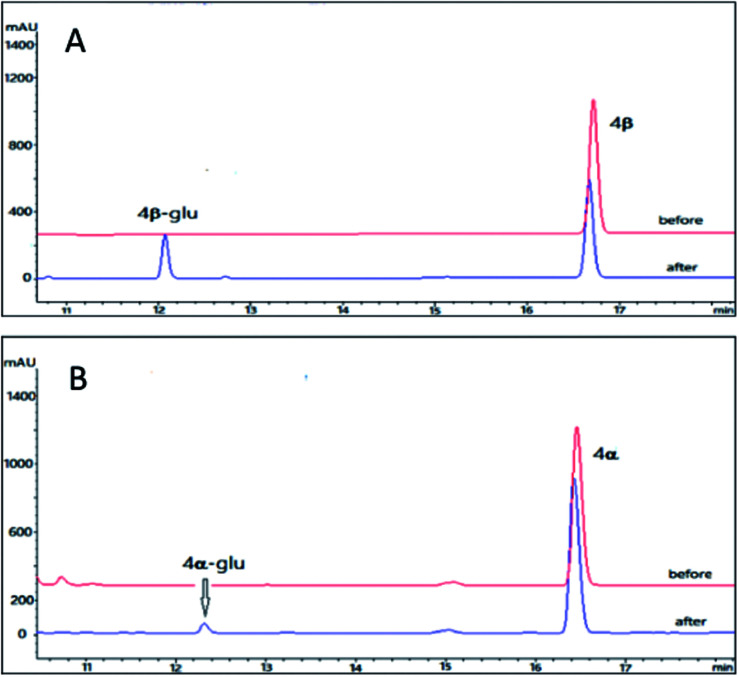
HPLC chromatograms of the enzymatic reactions. (A) 4β without UDPG and OleD (ASP) (before); 4β with UDPG and OleD (ASP) (after); (B) 4α without UDPG and OleD (ASP) (before); 4α with UDPG and OleD (ASP) (after).

Using the same reaction conditions as described for the bufadienolides, OleD (ASP) catalyzed the generation of monoglucoside of 4β-glu with a conversion rate of 26%; while the conversion rate for 4α is only 2% (Fig. 5). Thus the A/B ring fusion mode was also important for the conversion rate, and the lactone rings (either six-membered or five membered) at position C-17 did not affect the biotransformation.

We prepared the monoglucoside of 4β (4β-glu) for structural analysis. Compound 4β-glu (3.6 mg) was obtained by preparative HPLC with conversion rate 25% (10 mg 4β of was used as the substrate). Similar to the bufadienolide glycosides 1β-glu, 2β-glu and 3β-glu, the monoglucoside structure of 4β-glu was confirmed by ^1^D and ^2^D NMR and HR-ESI-MS analysis (*m*/*z* 537.8049 [M + H]^+^ C_29_H_44_O_9_, calcd for 537.8021). The glycosylation site was again determined at 3-OH by comparison of chemical shifts of C-3 (downfield 3.9 ppm) and H-3 (downfield 0.02 ppm) of 4β-glu with those of the parent compound 4β and the β-configuration of the glycosidic bond was confirmed by the large coupling constant of the anomeric proton (*δ*_H_ 4.32, d, *J* = 7.8 Hz). Accordingly, it was concluded that OleD (ASP) could catalyze the transformation of 4β into 4β-glu.

### The determination of *K*_m_ for the cardiotonic steroid epimers

As we can see that the substrate structure and stereochemistry of cardiotonic steroid epimers significantly affect the final glycosylation rate. In order to compare the dynamic process, *K*_m_ value was measured.

Epimers 2β and 2α representing the high conversion bufadienolides and 4β and 4α representing the cardenolides were chosen in this study. A series of concentrations of the substrates (0.01–1.2 mM) were incubated with the OleD (ASP) enzyme and UDPG, and the conversion rate was calculated by the corresponding HPLC peak area using the Agilent Chromatography Workstation Software. *K*_m_ value was determined based on the Michaelis–Menten equation.^25^ The result indicated that a high conversion rate corresponded to a low *K*_m_ value. Particularly, when the conversion rate is less than 2%, *K*_m_ value is toward a large value more than 100 mM (Table 2).

**Table 2:** Conversion rate and *K*_m_

Compound	Conversion^a^ (%)	*K*_m_ (mM)
2β	80	0.28 + 0.10
2α	1	>100
4β	26	0.81 + 0.14
4α	2	>100

a Conversion: calculated by the corresponding HPLC peak area percentage.

### Molecular modeling

**Fig. 6:**
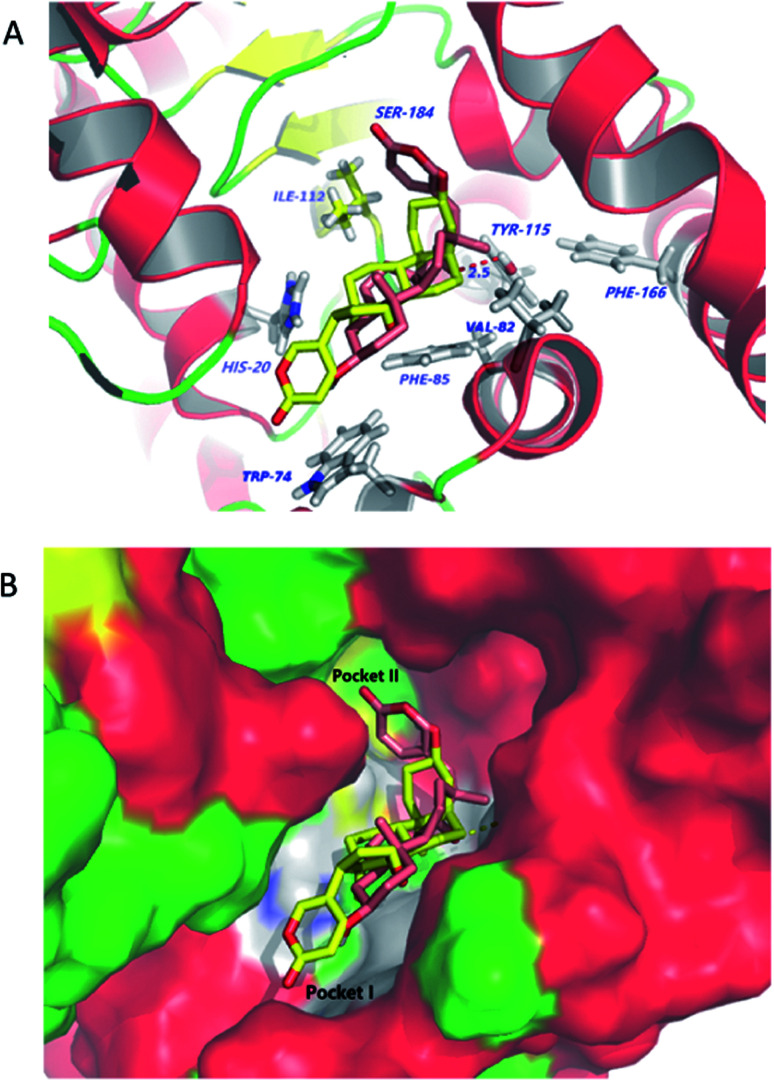
(A) Selected docking poses of compound 2α (depicted in yellow) and 2β (depicted in magenta) into the substrate cavity of OleD (ASP). The two ligands are shown in stick representation. The receptors are shown in cartoon representation with cyan alpha helices, green beta sheets and magenta loops. (B) OleD (ASP) (surface)–ligand (stick) complex.

Molecular docking studies shed new light on the mechanism of stereoselective glycosylation of cardiotonic steroids derivatives. Similar for the *K*_m_ study, we selected the high conversion enantiomers 2α and 2β to explore the binding mode in the enzyme substrate complex. As can be seen from Fig. 6A, the 2*H*-pyran-2-one ring of compound 2α penetrated deeply into the hydrophobic region I defined by His20, Phe85 and Trp74 residues. The epoxide contacted *via* van der Waals interactions with residues lle112 and Val82. In comparison to 2α binding to OleD (ASP), compound 2β showed an ‘inverse’ binding pose that C-3 aliphatic hydroxyl was placed in the hydrophobic pocket I and the 2*H*-pyran-2-one moiety bound in a hydrophobic cavity II (lle112, Ser184, Fig. 6B). Moreover, the oxygen atom of epoxide group formed a hydrogen bond (2.5 Å) with critical residue Tyr115. It can be inferred from docking results that the filling of hydrophobic pocket II and interacted with residue Tyr115 may play an important role in the *O*-linked glycosylation reactions catalyzed by OleD (ASP) glycosyltransferase, since the conversion efficiency is 1% for 2α and 80% for 2β, respectively.

### Inhibition of NKA activity

To explore the inhibitory activity of glycosylated products on NKA, the inhibition activity of CTS (1β, 2β, 3β, 4β) and their glycosylation products (1β-glu, 2β-glu, 3β-glu, 4β-glu) were determined using previous reported method.^11,27^ As seen in Table 3, glycosylation products showed a stronger inhibitory activity for NKA than the corresponding aglycones.

**Table 3:** Inhibition Na^+^, K^+^-ATPase activity

Compound	IC_50_ (μM)	Compound	IC_50_ (μM)
1β	1.15 ± 0.08	1β-glu	0.32 ± 0.03
2β	5.44 ± 0.48	2β-glu	1.94 ± 0.10
3β	3.43 ± 0.36	3β-glu	1.22 ± 0.10
4β	1.31 ± 0.02	4β-glu	0.78 ± 0.05

## Conclusions

In summary, this study demonstrated that the OleD-catalyze glycosylation of cardiotonic steroids are significantly influenced by the configuration at C-3 and the A/B fusion mode. 3β-OH and A/B ring *cis* fusion are favoured by OleD (ASP), while an acetyl group at C-16 and lactone ring type at C-17 did not influence the biotransformation. A high conversion rate corresponded to a low *K*_m_ value. Molecular docking simulation showed that filling the hydrophobic pocket II and interaction with residue Tyr115 may play an important role in the glycosylation reactions catalyzed by OleD (ASP) glycosyltransferase. Furthermore, the glycosylation products showed a stronger inhibitory activity for NKA than the corresponding aglycones. This study provided the first stereoselective properties for OleD (ASP) catalyzed glycosylation. It is noteworthy that glycosyltransferase has been used for the glycosylation of a wide variety of natural products;^40,41^ however, glycosyltransferase for the cardiotonic steroids is rare. Results of this study firstly revealed the stereo-selectivity of OleD-catalyzed glycosylation of cardiotonic steroids.

## Conflicts of interest

There are no conflicts to declare.

## Supplementary Material

RA-008-C7RA11979H-s001
