# Supplementary material for: Probing the stereoselectivity of OleD-catalyzed glycosylation of cardiotonic steroids
Source: RSC Adv. 2018 Jan 30;8(10):5071–8. doi: 10.1039/c7ra11979h (PMC9078122; doi:10.1039/c7ra11979h)

Electronic Supplementary Material (ESI) for RSC Advances.
This journal is © The Royal Society of Chemistry 2018

Supporting Information for:

Probing the Stereoselectivity OleD-Catalyzed Glycosylation with Cardiotonic Steroids

Xue-Lin Zhu[1⊥], Chao Wen[1⊥], Qing-Mei Ye[2⊥], Wei Xu[1], Deng-Lang Zou[1], Guang-Ping Liang[1], Fan Zhang[1], Wan-Na Chen[1], Ren-Wang Jiang[1*]

[1] *Guangdong Province Key Laboratory of Pharmacodynamic Constituents of TCM and New Drugs Research, College of Pharmacy, Jinan University, Guangzhou 510632, P. R. China.*

[2] *Department of Pharmacy, Hainan General Hospital, Haikou 570311, P. R. China.*

[⊥] *These authors contribute equally to this work.*

[*] *Ren-Wang Jiang, e-mail: trwjiang@jnu.edu.cn (R.W.J.)*

Table of Content

1. Determination of kinetic parameters

2. Assessment of inhibition of NKA activity by CTS

3. Spectral data and characterization of **1α, 2α, 3α, 1β-glu, 2β-glu, 2β-diglu, β-glu, 3β-diglu, 4β-glu.**

4. Spectra of compounds

1. Determination of kinetic parameters.

Assays were performed in a final volume of 200 μL 50 mM Tris-HCl (pH 8.0), and contained constant concentrations of OleD (ASP) (40 μg) and UDPG (2.5 mM) while varying the concentration (0.01-1.2 mM) of **2β**, **2α**, **4β** and **4α**. Aliquots (100 μL) were removed every 15 min, mixed with an equal volume of ice cold mathanol, and centrifuged at 10,000 g for 10 min. Supernatants were analyzed by analytical reverse-phase HPLC. Conversion rate is calculated by the corresponding HPLC peak area percentage using the Aglient Chromatography Workstation Software. All experiments were performed in triplicate. Initial velocities were fitted to the Michaelis-Menten equation using Origin Pro 7.0 software.

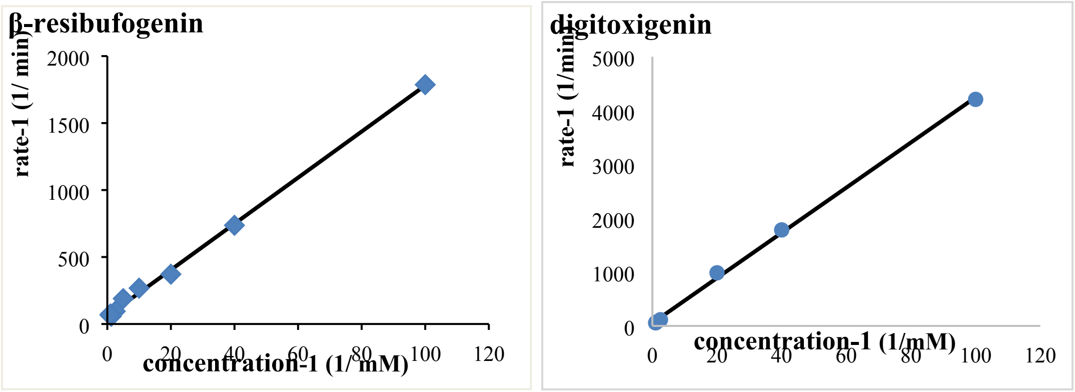

Figure S1. The Determination of K_m for the **2β** and **4β**.

2. Assessment of inhibition of NKA activity by CTS

NKA from pig kidney microsomal membranes was purified by differential centrifugation as reported. Stock solutions of the inhibitors, typically 10 mM, were prepared in DMSO. In brief, NKA is preincubated at 37 °C for 2 hours in the presence of 3 mM $MgCl_2$, 3 mM Na-phosphate and 40 mM Tris-HCl (pH 7.0) with increasing

concentrations of inhibitor. The residual NKA activity is subsequently determined by 40-fold dilution into a standard ATPase assay medium in triplicate. For each inhibitor of concentration the residual activity was determined in three independent experiments.

The maximal DMSO concentration of 2.5% after dilution into the NKA incubation medium was 2.5%. Control experiments showed that incubation with 2.5% DMSO for 2 hours at 37 °C leads to less than 10% inhibition of NKA activity.

3. Spectral data and characterization of 1α, 2α, 3α, 1β-glu, 2β-glu, 2β-diglu, β-glu, 3β-diglu, 4β-glu.

1α: ^{1}H NMR (CD_3OD, 400 MHz) *δ*: 8.01 (1H, dd, *J* = 9.7, 2.5 Hz, H-22), 7.45 (1H, d, *J* = 2.4 Hz, H-21), 6.30 (1H, d, *J* = 9.8 Hz, H-23), 3.58 (1H, m, H-3), 2.58 (1H, m, H-17), 2.26-2.11 (2H, m), 1.92-1.62 (11H, m), 1.55-1.36 (9H, m), 1.29-1.19 (1H, m), 1.09-1.01 (2H, m), 0.95 (3H, s, H-19), 0.73 (3H, s, H-18); ^{13}C NMR (CD_3OD, 100 MHz) *δ*: 164.8 (C-24), 150.5 (C-21), 149.37 (C-22), 125.0 (C-20), 115.4 (C-23), 86.1 (C-14), 72.3 (C-3), 52.3 (C-17), 49.8 (C-13), 43.2 (C-9), 43.1 (C-5), 41.9 (C-12), 37.7 (C-8), 37.0 (C-4), 36.3 (C-1), 35.9 (C-10), 33.2 (C-15), 31.3 (C-2), 29.9 (C-16), 28.4 (C-6), 23.8 (C-19), 22.8 (C-7), 22.5 (C-11), 17.3 (C-18). HRESIMS m/z 386.5343 $[M + H]^+$ (calcd for $C_{24}H_{34}O_4$, 386.5541).

In the ^{1}H-NMR spectrum of **1α**, the chemical shift of hydrogen at C-3 resonates at *δ* 3.65 with a multiplet pattern, which indicates that the hydroxyl group should be *α*-oriented because the *α*-oriented proton H-3, adopting the axial position, is split by two axial protons (H-2*α* and H-4*α*) and two equatorial proton (H-2β and H-4β) resulting large *aa* and small *ae* couplings. In contrast, in the ^{1}H-NMR spectrum of **1β**, the

hydrogen at C-3 resonates at *δ* 4.13with a broad singlet pattern.

2α: ^{1}H NMR (CD_3OD, 400 MHz) *δ*: 7.91 (1H, dd, *J* = 9.8, 2.4 Hz, H-22), 7.47 (1H, d, *J* = 2.4 Hz, H-21), 6.28 (1H d, *J* = 9.8 Hz, H-23), 3.62 (1H, s, H-15), 3.57 (1H, m, H-3), 2.61 (1H, d, *J* = 10.0 Hz, H-10), 2.44 (1H, d, *J* = 10.4 Hz, H-16), 2.04-1.63 (9H, m), 1.63-1.28 (9H, m), 0.99 (3H, s), 0.79 (3H, s); ^{13}C NMR (CD_3OD, 100 MHz) *δ*: 164.5 (C-24), 151.8 (C-21), 149.6 (C-22), 124.5 (C-20), 115.3 (C-23), 75.8 (C-14), 72.2 (C-3), 61.1 (C-15), 48.6 (C-17), 46.3 (C-13), 43.1 (C-5), 41.3 (C-9), 40.1 (C-12), 36.9 (C-4), 36.1 (C-1), 36.0 (C-10), 35.2 (C-8), 33.2 (C-16), 31.3 (C-2), 27.5 (C-6), 23.8 (C-19), 22.1 (C-11), 22.0 (C-7), 17.1 (C-18). HRESIMS m/z 385.5243 $[M + H]^{+}$ (calcd for $C_{24}H_{32}O_4$, 385.5241).

In the ^{1}H-NMR spectrum of **2α**, the chemical shift of hydrogen at C-3 resonates at *δ* 3.57 with a multiplet pattern, which indicates that the hydroxyl group should be *α*-oriented because the *α*-oriented proton H-3, adopting the axial position, is split by two axial protons (H-2*α* and H-4*α*) and two equatorial proton (H-2β and H-4β) resulting large *aa* and small *ae* couplings. In contrast, in the ^{1}H-NMR spectrum of **2β**, the hydrogen at C-3 resonates at *δ* 4.06 with a broad singlet pattern.

3α : ^{1}H NMR (CD_3OD, 400 MHz) *δ*: 8.04 (1H, d, *J* = 9.6 Hz, H-22), 7.39 (1H, s, H-21), 6.26 (1H, d, *J* = 9.6 Hz, H-23), 5.51 (1H, d, *J* = 10.3 Hz, H-16), 3.76 (1H, s, H-15), 3.58 (1H, m, H-3,), 2.95 (1H, d, *J* = 9.3 Hz, H-17), 2.20-2.01 (1H, td, H-8), 1.81(3H, s, $COCH_3$,) 1.79-1.04 (15H, m,), 0.99 (3H, s, H-19), 0.83 (3H, s, H-18); ^{13}C NMR (CD_3OD, 100 MHz) *δ*: 171.6 ($COCH_3$), 164.0 (C-24), 153.5 (C-22), 150.9 (C-21), 118.4 (C-20), 114.1 (C-23), 76.6 (C-16), 73.4 (C-14), 72.2 (C-3), 60.8 (C-15),

51.4 (C-17), 46.3 (C-13), 43.0 (C-5), 41.1 (C-9), 40.7 (C-12), 36.9 (C-4), 36.1 (C-1), 36.0 (C-10), 34.7 (C-8), 31.2 (C-2), 27.4(C-6), 23.7 (C-19), 21.9 (C-7), 21.9 (C-11), 20.4 ($COCH_3$), 17.5 (C-18). HRESIMS m/z 443.4345 $[M + H]^+$ (calcd for $C_{26}H_{34}O_6$, 443.5134).

In the ^{1}H-NMR spectrum of **3α**, the chemical shift of hydrogen at C-3 resonates at *δ* 3.58 with a multiplet pattern, which indicates that the hydroxyl group should be *α*-oriented because the *α*-oriented proton H-3, adopting the axial position, is split by two axial protons (H-2*α* and H-4*α*) and two equatorial proton (H-2β and H-4β) resulting large *aa* and small *ae* couplings. In contrast, in the ^{1}H-NMR spectrum of **3β**, the hydrogen at C-3 resonates at *δ* 4.07 with a broad singlet pattern.

1β-glu : ^{1}H NMR (CD_3OD, 400 MHz) *δ*: 8.03 (1H, dd, *J* = 9.7, 2.3 Hz, H-22), 7.45 (1H, d, *J* = 1.8 Hz, H-21), 6.30 (1H, d, *J* = 9.6 Hz, H-23), 4.37 (1H, d, *J* = 7.8 Hz, sugar H-1), 4.12 (1H, m, H-3), 3.88 (1H, d, *J* = 11.9, 1.8 Hz), 3.71 (1H, dd, *J* = 5.4 Hz), 3.40-3.20 (4H, m), 2.56-2.52 (1H, m, H-17), 2.26-2.11 (2H, m), 1.92-1.22 (23H, m), 1.09-1.01 (2H, m), 0.95 (3H, s, H-19), 0.73 (3H, s, H-18); ^{13}C NMR (CD_3OD, 100 MHz) *δ*: 164.8 (C-24), 150.5 (C-21), 149.37 (C-22), 125.0 (C-20), 115.4 (C-23), 102.7 (sugar C-1), 86.1 (C-14), 78.2 (sugar C-5), 77.84 (sugar C-2), 75.6 (sugar C-4), 75.2 (sugar C-3), 71.8 (C-3), 61.7 (sugar C-6), 52.3 (C-17), 49.8 (C-13), 43.2 (C-9), 43.1 (C-5), 41.9 (C-12), 37.7 (C-8), 37.0 (C-4), 36.3 (C-1), 35.9 (C-10), 33.2 (C-15), 31.3 (C-2), 29.9 (C-16), 28.4 (C-6), 23.8 (C-19), 22.8 (C-7), 22.5 (C-11), 17.3 (C-18). HRESIMS m/z 549.3035 $[M + H]^+$ (calcd for $C_{30}H_{44}O_9$, 549.3021).

2β-glu: ^{1}H NMR (CD_3OD, 400 MHz) *δ*: 7.91 (1H, dd, *J* = 9.8, 2.4 Hz, H-22), 7.46

(1H, d, *J* = 2.4 Hz, H-21), 6.27 (1H d, *J* = 9.8 Hz, H-23), 4.32 (1H, d, *J* = 7.8 Hz, sugar H-1), 4.08 (1H, m, H-3), 3.86 (1H, d, *J* = 11.9 Hz), 3.67 (1H, dd, *J* = 11.9, 5.4 Hz), 3.62 (1H, s, H-15), 3.39-3.17 (7H, m), 2.61 (1H, d, *J* = 10.0 Hz, H-10), 2.44 (1H, d, *J* = 10.4 Hz, H-16), 2.04-1.43 (15H, m), 1.40-1.05 (4H, m), 0.99 (3H, s), 0.79 (3H, s); ^{13}C NMR (CD_3OD, 100 MHz) δ: 164.5 (C-24), 151.8 (C-21), 149.6 (C-22), 124.5 (C-20), 115.3 (C-23), 102.7 (sugar C-1), 78.2 (sugar C-5), 77.84 (sugar C-2), 75.8 (C-14), 75.4 (sugar C-4), 75.2 (sugar C-3), 71.7 (C-3), 62.8 (sugar C-6), 61.2 (C-15), 48.6 (C-17), 46.3 (C-13), 40.7 (C-12), 40.1 (C-9), 37.4 (C-10), 36.3 (C-5), 35.0 (C-4), 33.2 (C-8), 31.1 (C-1), 30.8 (C-16), 27.5 (C-2), 27.0 (C-6), 24.1 (C-19), 22.2 (C-11), 21.7 (C-7), 17.2 (C-18). HRESIMS m/z 547.2902 [M + H] $^+$ (calcd for $C_{30}H_{42}O_9$, 547.2938).

2β-diglu: HRESIMS m/z 709.3421 [M + H] $^+$.

3β-glu : ^{1}H NMR (CD_3OD, 400 MHz) δ: 7.91 (1H, dd, *J* = 9.8, 2.4 Hz, H-22), 7.47 (1H, d, *J* = 2.4 Hz, H-21), 6.28 (1H d, *J* = 9.8 Hz, H-23), 4.34 (1H, d, *J* = 7.8 Hz, sugar H-1), 4.10 (1H, m, H-3), 3.87 (1H, dd, *J* = 11.8, 1.8 Hz), 3.76 (1H, s, H-15), 3.69 (1H, dd, *J* = 11.8, 5.4 Hz), 3.58 (1H, m, H-3), 3.45-3.17 (5H, m), 2.95 (1H, d, *J* = 9.3 Hz, H-17), 2.15-2.01 (1H, td, H-8), 1.85(3H, s, $COCH_3$,) 1.81-1.04 (19H, m,), 0.99 (3H, s, H-19), 0.83 (3H, s, H-18); ^{13}C NMR (CD_3OD, 100 MHz) δ: 171.6 ($COCH_3$), 164.0 (C-24), 153.5 (C-22), 150.9 (C-21), 118.4 (C-20), 114.1 (C-23), 102.7 (sugar C-1), 78.2 (sugar C-5), 77.84 (sugar C-2), 76.6 (C-16), 75.4 (sugar C-4), 75.2 (sugar C-3), 73.4 (C-14), 71.7 (C-3), 62.8 (sugar C-6), 60.8 (C-15), 51.4 (C-17), 46.3 (C-13), 40.5 (C-5), 40.7 (C-9), 37.3 (C-12), 36.3 (C-4), 36.1 (C-1), 36.0 (C-10),

31.7 (C-8), 31.2 (C-2), 27.4 (C-6), 23.7 (C-19), 21.9 (C-7), 21.9 (C-11), 20.4 ($COCH_3$), 17.5 (C-18). HRESIMS m/z 605.2996 [M + H] $^+$ (calcd for $C_{32}H_{44}O_{11}$, 605.2938).

3β-diglu：HRESIMS m/z 767.3479 [M + H] $^+$.

4β-glu: ^{1}H NMR (CD_3OD, 400 MHz) *δ*: 5.93 (1H, s), 5.07 (1H, d, *J* = 18.4 Hz), 4.95 (1H, d, *J* = 19.8 Hz), 4.35 (1H, d, *J* = 7.8 Hz, sugar H-1), 4.11 (1H, s, H-3), 3.89 (1H, d, *J* = 2.1 Hz), 3.69 (1H, dd, *J* = 11.8, 5.4 Hz), 3.40-3.21 (5H, m), 2.95-2.79 (1H m,), 2.35-2.12 (2H, m), 2.00-1.43 (19H, m), 1.40-1.22 (4H, m), 1.00 (3H, s), 0.92 (3H, s); ^{13}C NMR (CD_3OD, 100 MHz) *δ*: 178.5 (C-23), 177.3 (C-20), 117.8 (C-22), 102.7 (sugar C-1), 86.5 (C-14), 78.2 (sugar C-5), 77.8 (sugar C-2), 75.4 (sugar C-4), 75.3 (sugar C-3), 75.2 (C-31), 71.7 (C-3), 62.8 (sugar C-6), 52.2 (C-13), 51.1 (C-17), 42.7 (C-9), 41.0 (C-8), 37.5(C-5), 36.9 (C-12), 36.3 (C-10), 33.4(C-4), 31.2 (C-15), 30.9 (C-1), 28.1 (C-2), 27.8 (C-6), 27.5 (C-16), 24.1 (C-7), 22.6 (C-7), 22.4 （C-18）, 16.4 (C-19). HRESIMS m/z 537.8049 [M + H] $^+$ (calcd for $C_{29}H_{44}O_9$, 537.8021).

4. Spectra of compounds

Figure S2. 1α of HR-MS

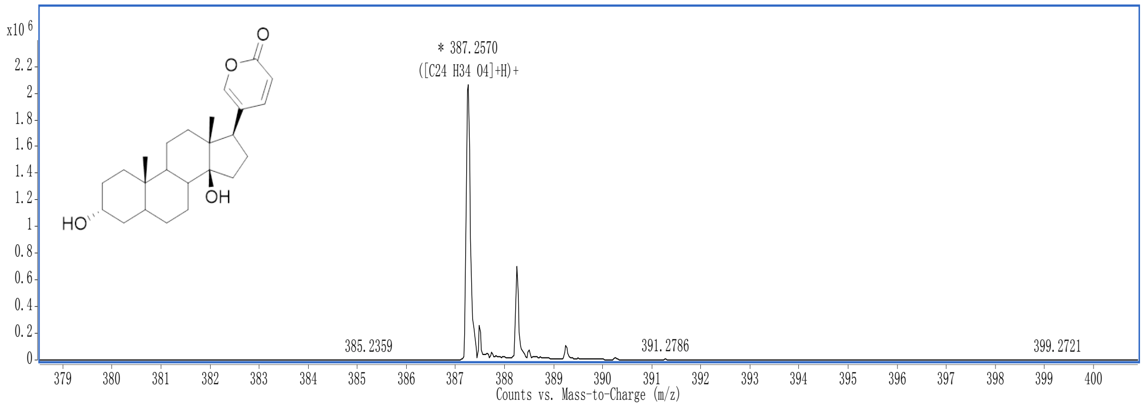

Figure S3. ^{1}H (CD_3OD, 400MHz)

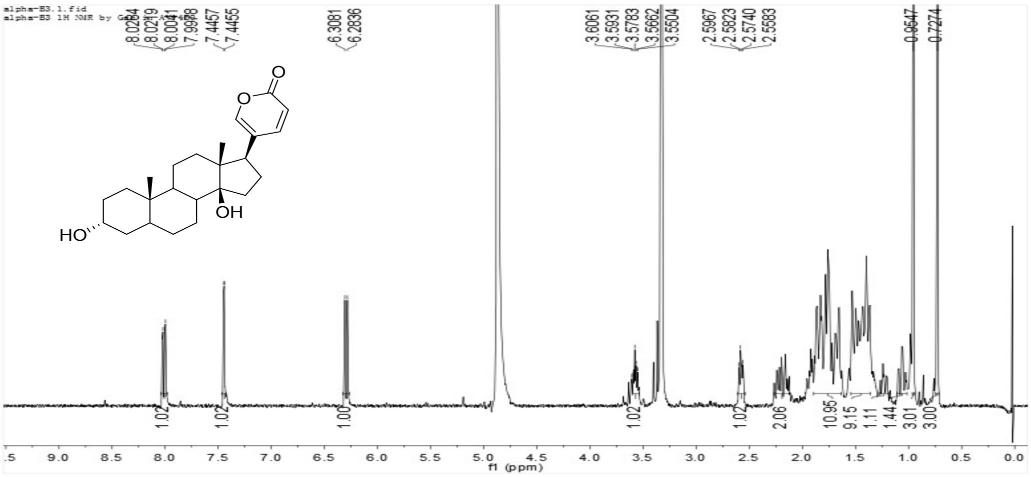

Figure S4. ^{13}C (CD_3OD, 100MHz)

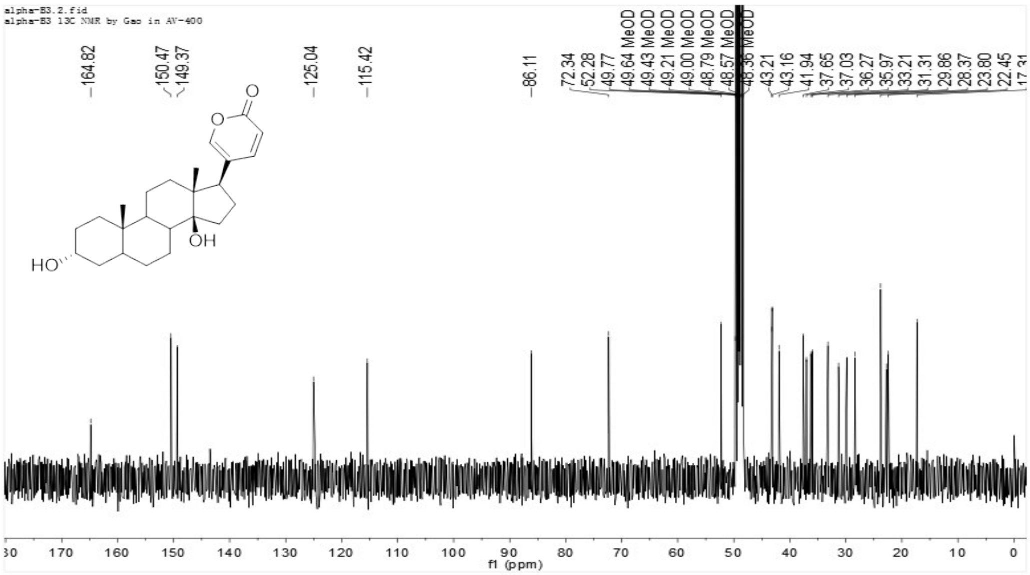

Figure S5. 2α of HR-MS

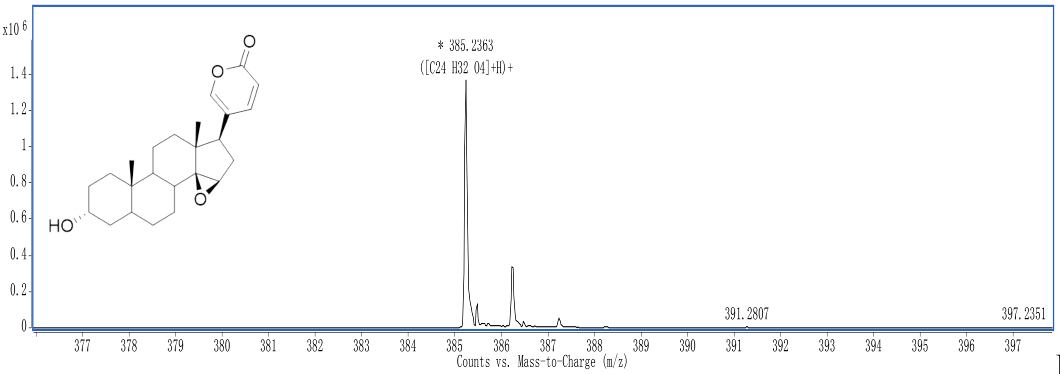

Fi

gure S6. ^{1}H (CD$_3$OD, 400MHz)

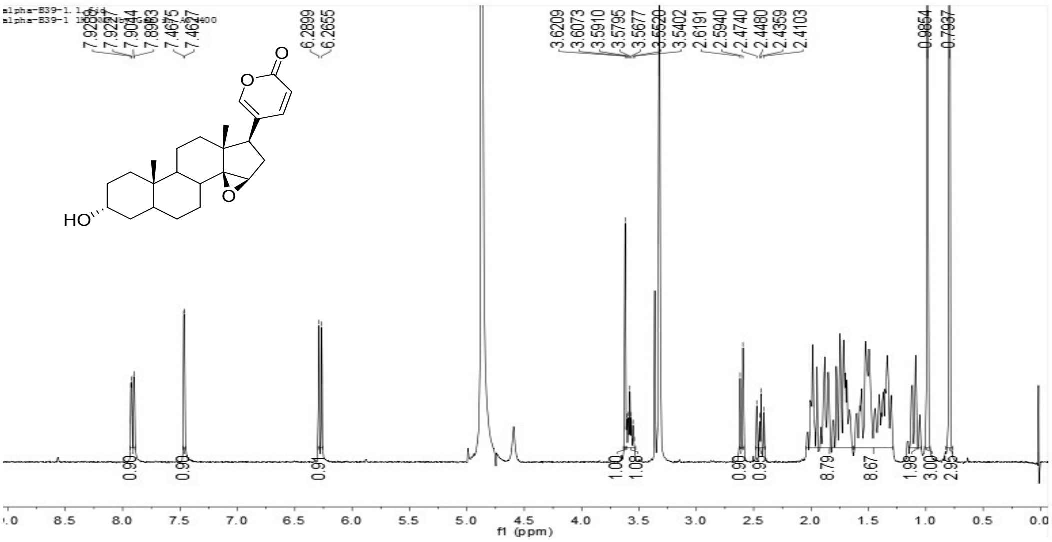

Figure S7. ^{13}C (CD$_3$OD, 100MHz)

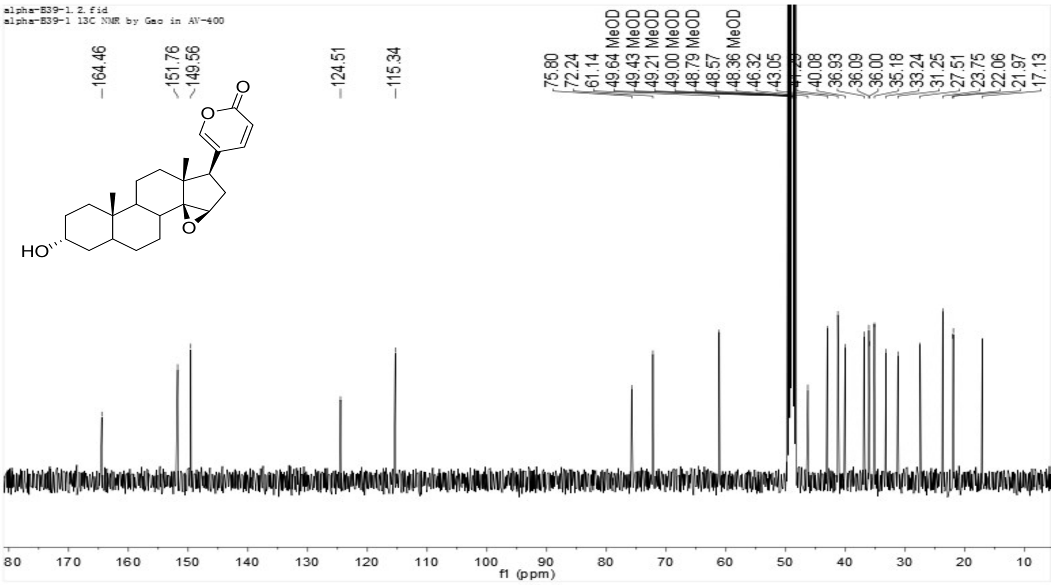

Figure S8. 3α of HR-MS

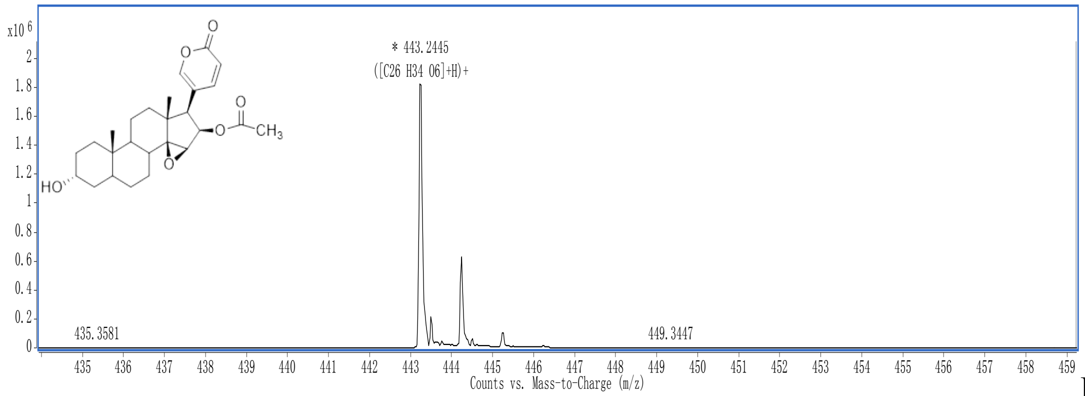

F

igure S9. ^{1}H (CD_3OD, 400MHz)

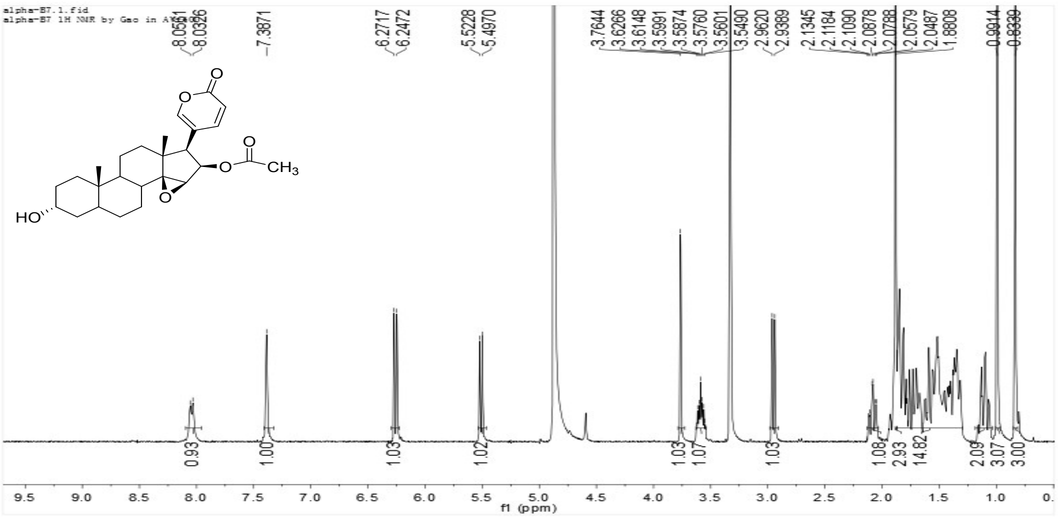

Figure S10. ^{13}C (CD_3OD, 100MHz)

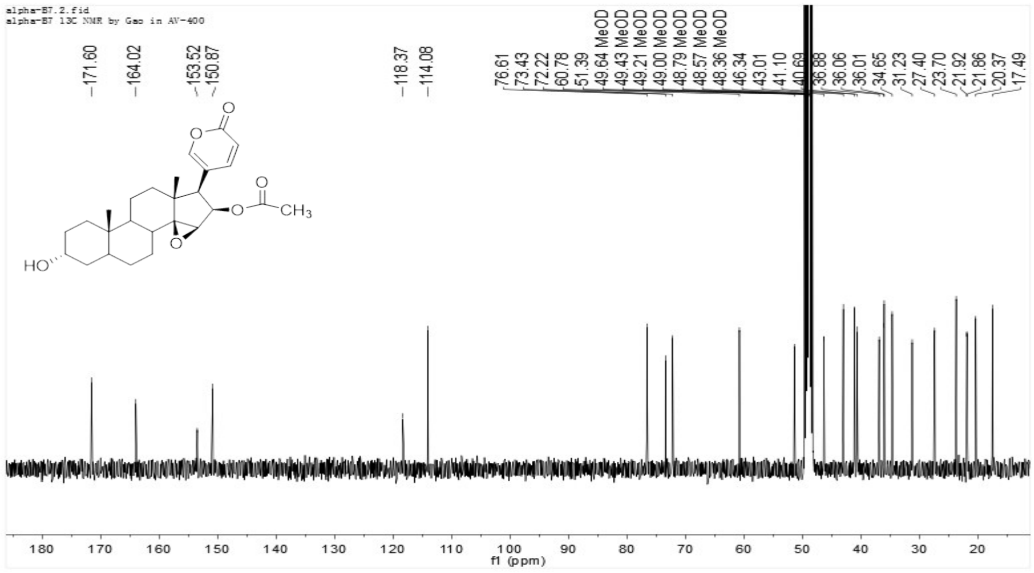

Figure S11. 1β-glu of HR-MS

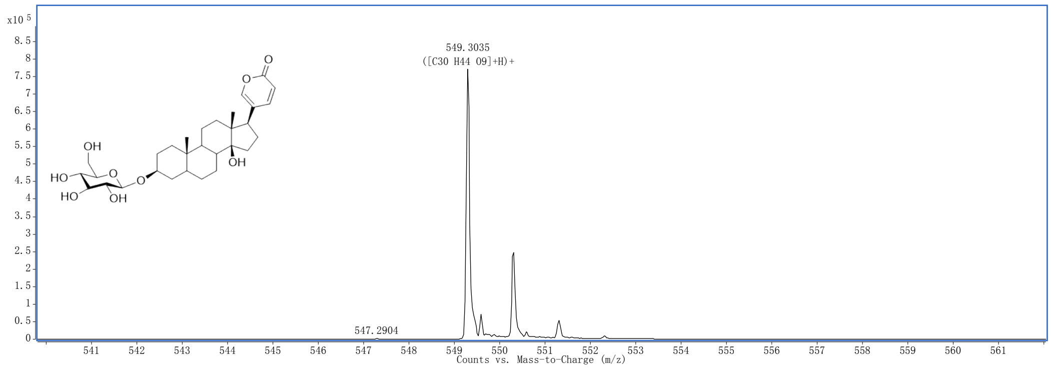

Figure S12. ^{1}H (CD_3OD, 400MHz)

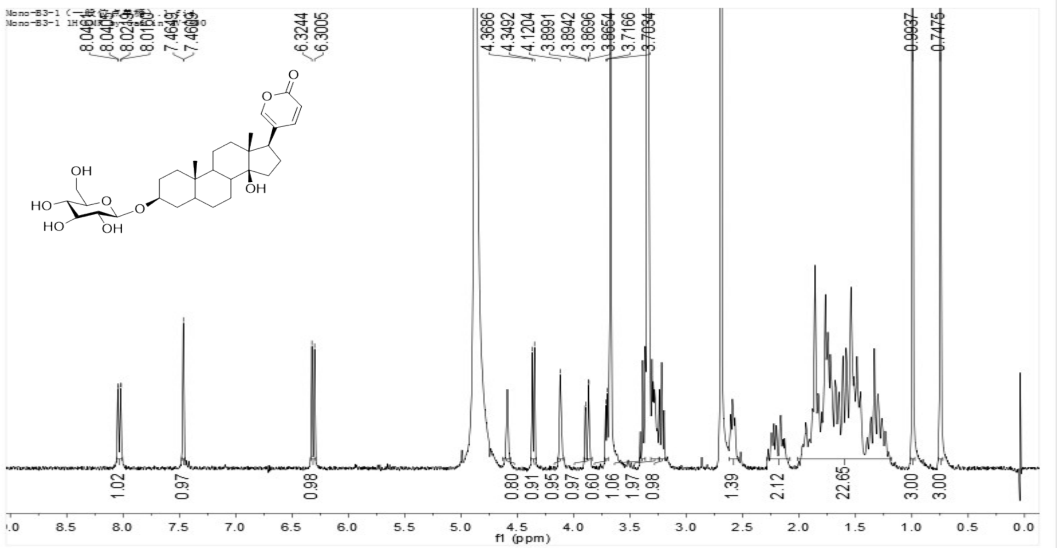

Figure S13. ^{13}C (CD_3OD, 100MHz)

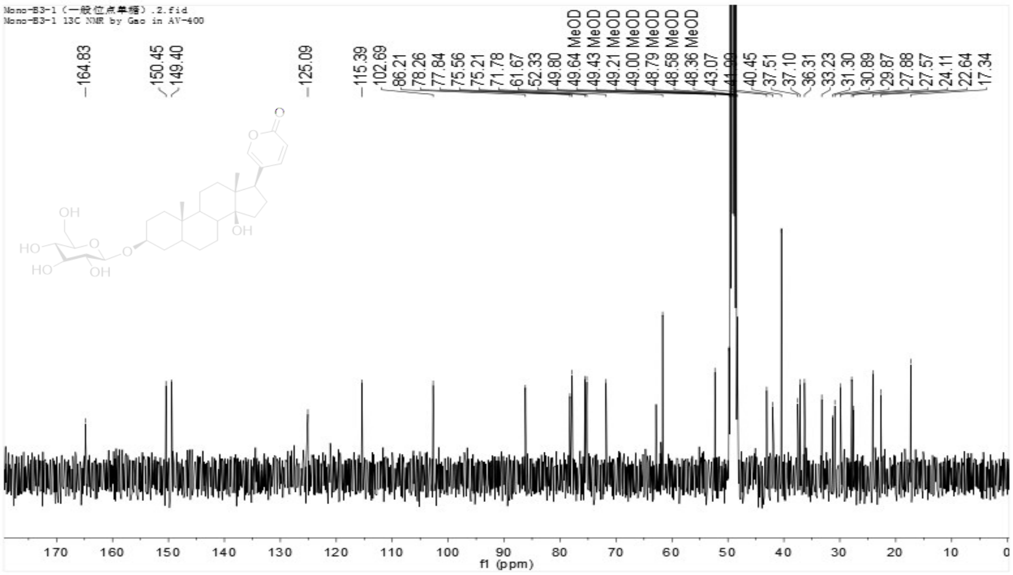

Figure S14. HSQC (CD_3OD, 400MHz)

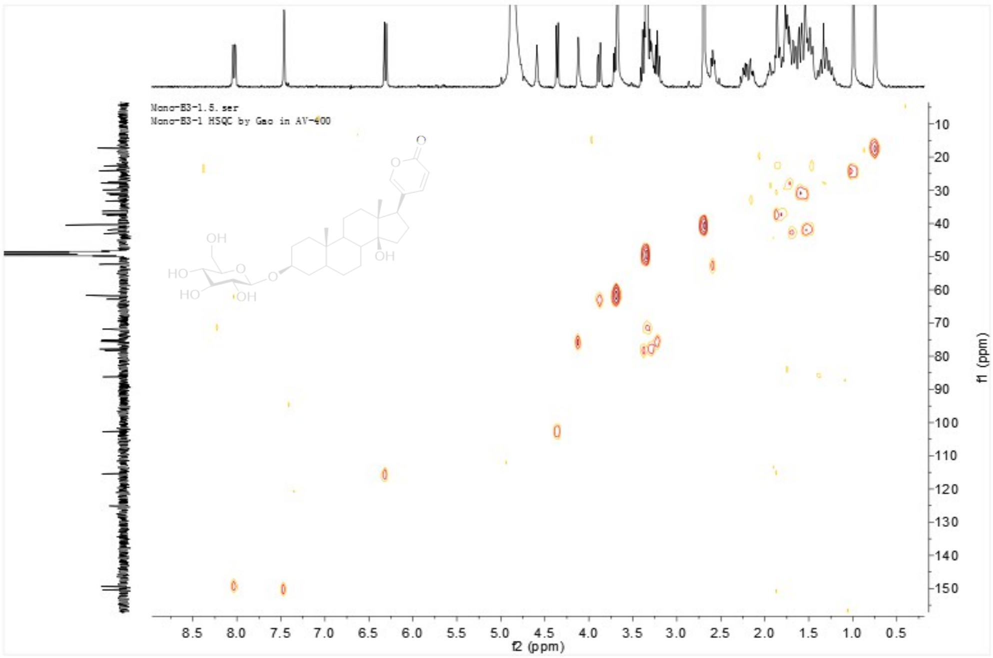

Figure S15. 2β-glu of HR-MS

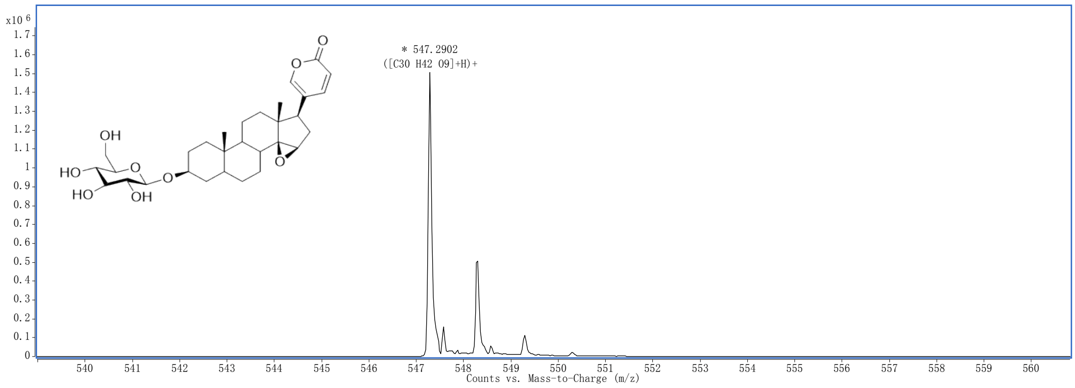

Figure S16. ^1H (CD_3OD, 400MHz)

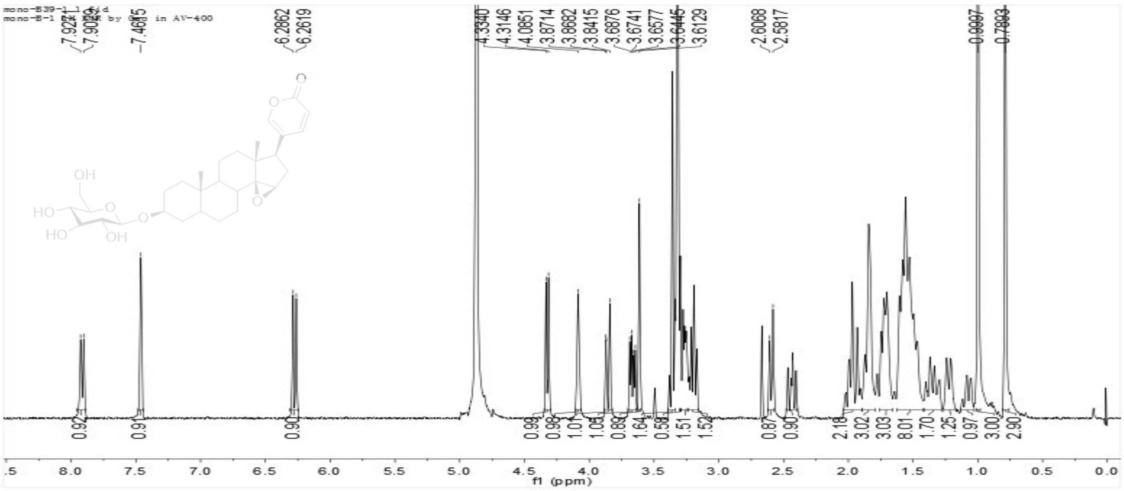

Figure S17. ^{13}C (CD_3OD, 100MHz)

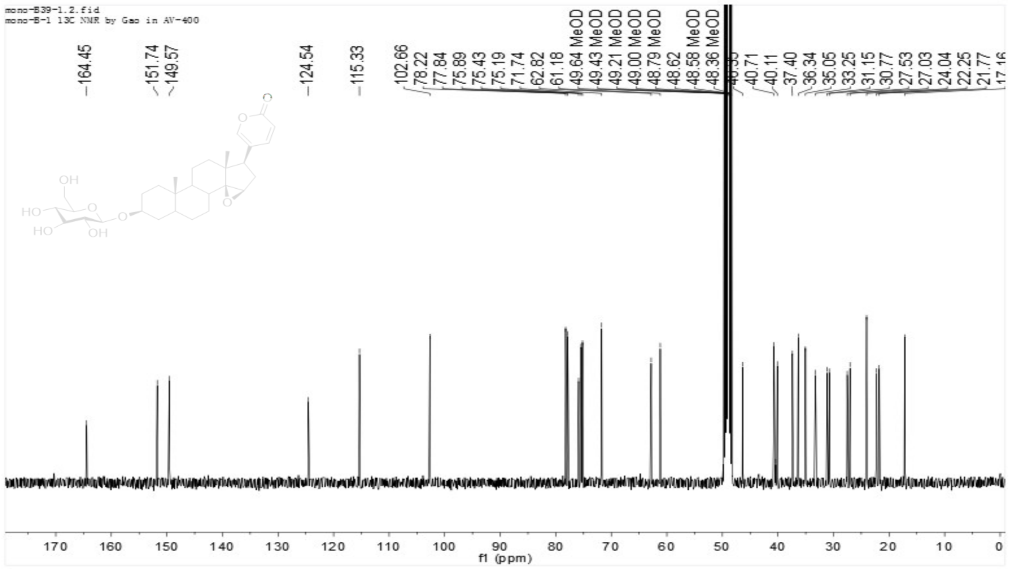

Figure S18. HSQC (CD_3OD, 400MHz)

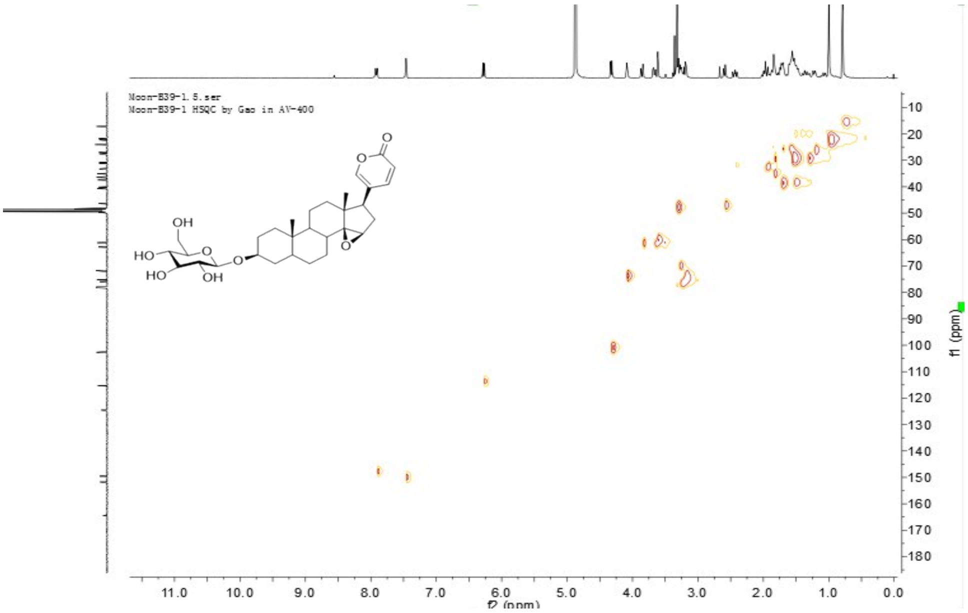

Figure S19. 2β-diglu of HR-MS

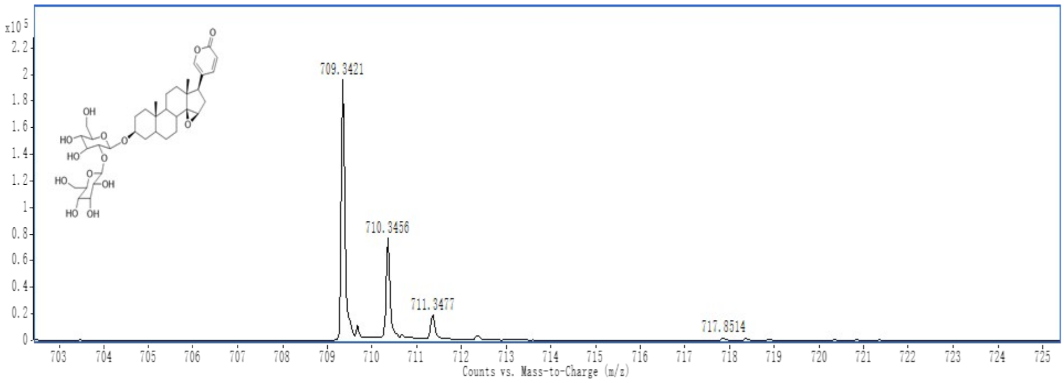

Figure S20. 3β-glu of HR-MS

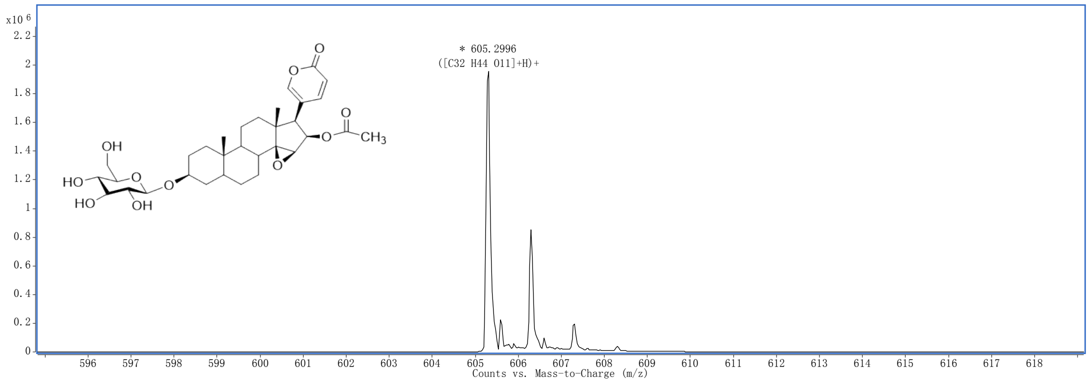

Figure S21. ^{1}H (CD_3OD, 400MHz)

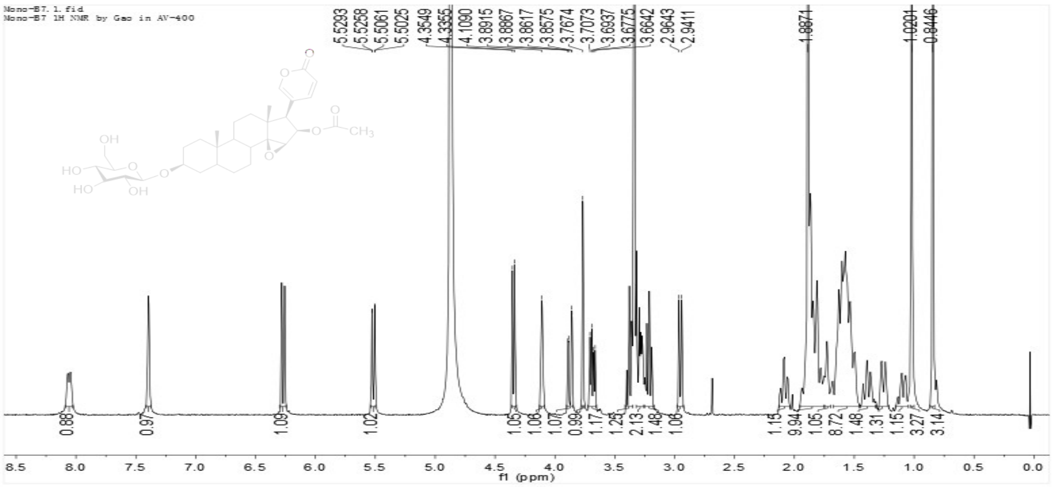

Figure S22. ^{13}C (CD_3OD, 100MHz)

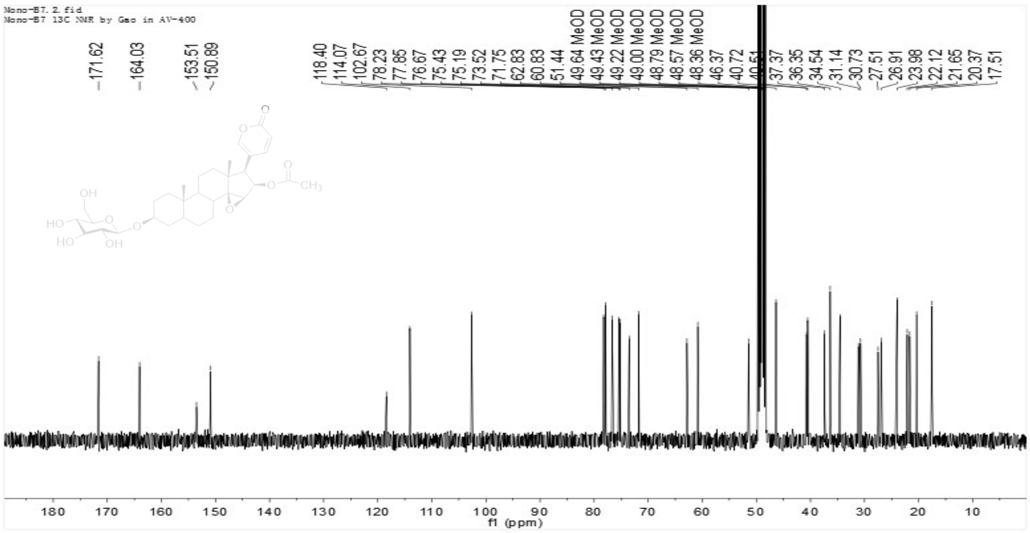

Figure S23. HSQC (CD_3OD, 400MHz)

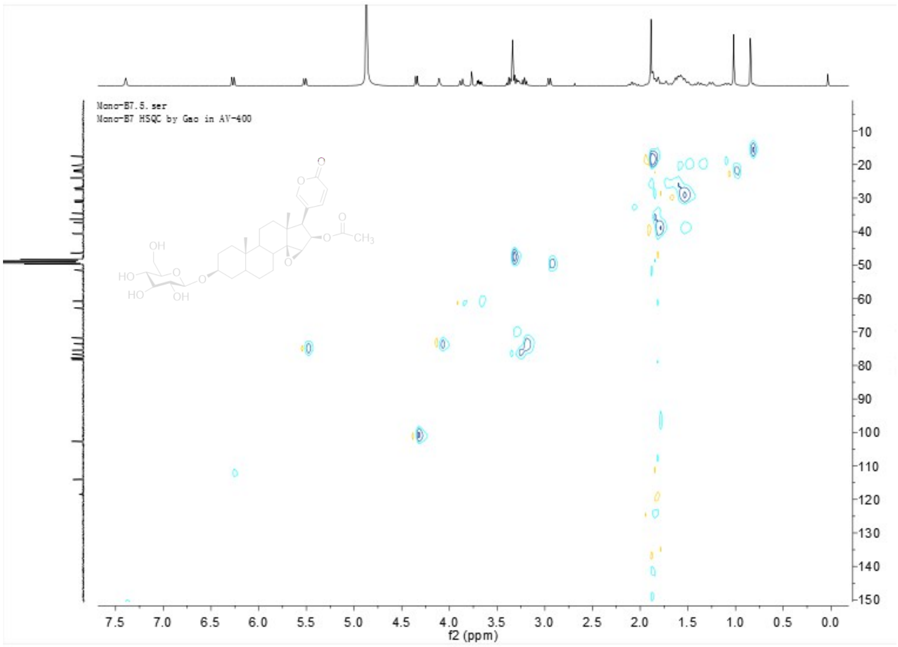

Figure S24. 3β-diglu of HR-MS

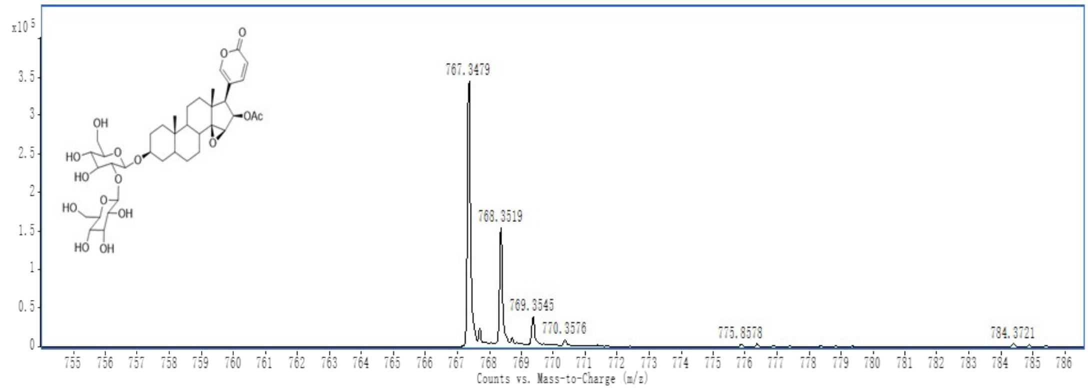

Figure S25. 4β-glu of HR-MS

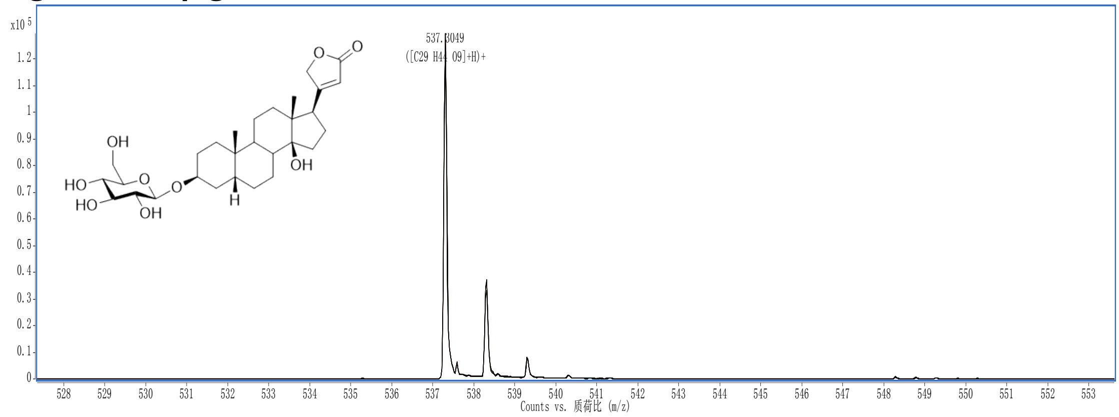

Figure S26. ${}^{1}H$ (CD_3OD, 400MHz)

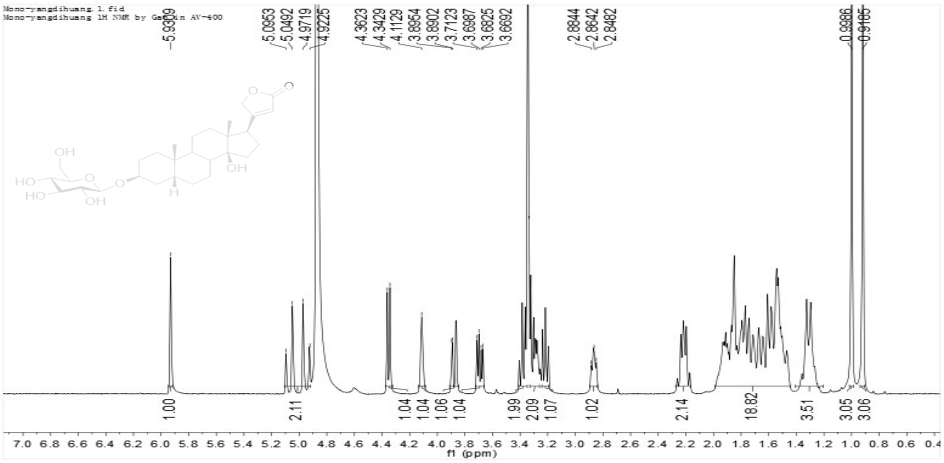

Figure S27. ^{13}C (CD_3OD, 100MHz)

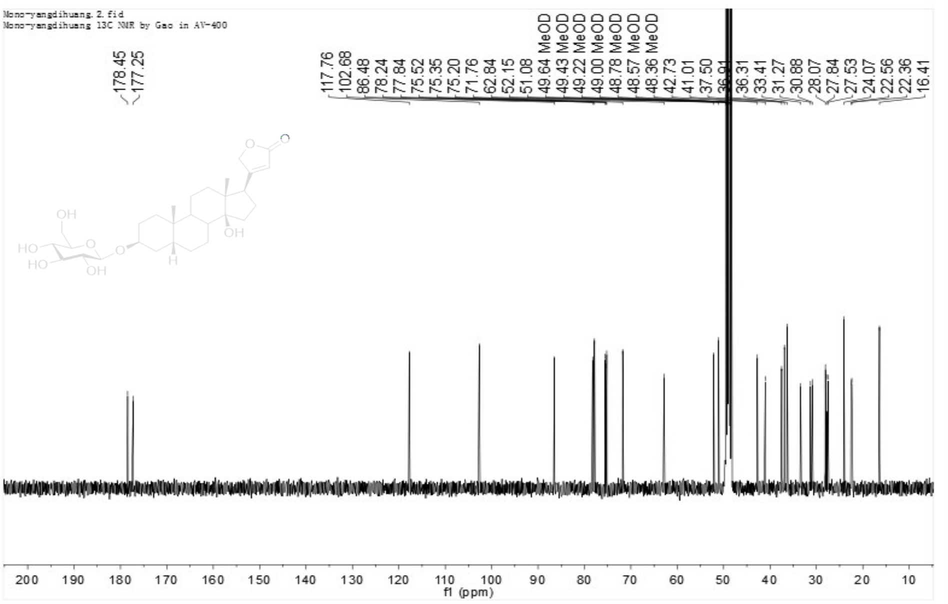

Figure S28. HSQC (CD_3OD, 400MHz)

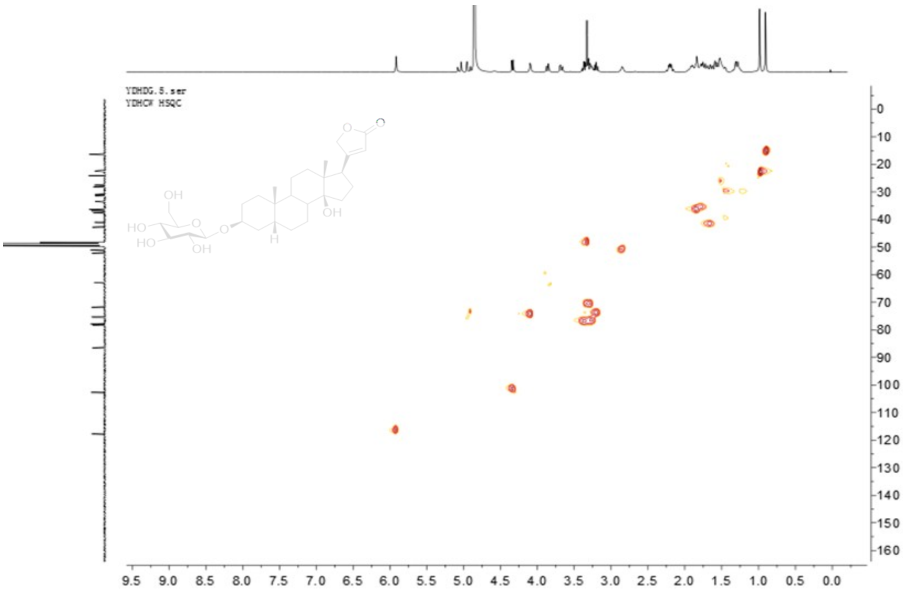

Supplement: RA-008-C7RA11979H-s001 [file RA-008-C7RA11979H-s001.pdf]
